# Interaction of Fear Conditioning with Eyeblink Conditioning Supports the Sensory Gating Hypothesis of the Amygdala in Men

**DOI:** 10.1523/ENEURO.0128-20.2020

**Published:** 2020-09-23

**Authors:** Lana Inoue, Thomas Michael Ernst, Inda Inat Ferber, Christian Josef Merz, Dagmar Timmann, Giorgi Batsikadze

**Affiliations:** 1 Department of Neurology, Essen University Hospital, University of Duisburg-Essen, 45147 Essen, Germany; 2 Department of Biotechnology, Tel Hai College, Tel Hai 1220800, Israel; 3 Faculty of Psychology, Institute of Cognitive Neuroscience, Department of Cognitive Psychology, Ruhr University Bochum, 44780 Bochum, Germany

**Keywords:** amygdala, cerebellum, eyeblink conditioning, fear conditioning, fear extinction

## Abstract

Inhibition of the amygdala slows down acquisition of conditioned eyeblink responses (CRs). Based on the two-stage or two-factor theory of aversive conditioning, amygdala-dependent conditioned fear is a necessary prerequisite to acquire eyeblink CRs but is no longer needed after eyeblink CRs are attained. According to the sensory gating hypothesis of the amygdala, on the other hand, the amygdala modulates the salience of unconditioned stimuli (USs) and conditioned stimuli (CSs) in eyeblink conditioning. We tested these two opposing assumptions in five groups of 20 young and healthy men. On day 1, three groups underwent fear acquisition training followed by acquisition of eyeblink CRs. On the next day (day 2), extinction was tested. In group 1, fear and eyeblink extinction trials overlapped; in group 2, fear and eyeblink extinction trials alternated; and in group 3, fear extinction trials were followed by eyeblink extinction trials. Groups 4 and 5 were control conditions testing fear and eyeblink conditioning only. Preceding fear acquisition training facilitated acquisition of conditioned eyeblinks. Concomitant fear extinction impeded extinction of eyeblink CRs, which was accompanied by increased autonomic responses. Fear extinction, however, was not significantly altered by concomitant eyeblink extinction. Recall of fear CRs on day 2 was facilitated in group 1, suggesting additive response summation. Findings are difficult to explain with the two-stage theory of aversive conditioning, which predicts the suppression of conditioned fear once conditioned eyeblinks are acquired. Facilitated acquisition and impeded extinction of eyeblink CRs, however, are in accordance with the sensory-gating hypothesis of the amygdala.

## Significance Statement

It has been proposed that conditioned eyeblink responses, once established, may help to facilitate fear extinction. This has potential clinical relevance because the extinction of learned fear responses is at the core of exposure therapy in the treatment of many anxiety disorders. Based on our findings, this proposal has to be rejected. Our findings do not support the two-stage theory of aversive conditioning, which predicts the suppression of conditioned fear once conditioned eyeblinks are acquired. Rather, we found that concomitant extinction of conditioned eyeblink and fear responses facilitated the recall of conditioned fear responses and impeded the extinction of conditioned eyeblinks. Findings are best explained by increased salience of conditioned stimuli and, therefore, support the sensory-gating hypothesis of the amygdala.

## Introduction

Eyeblink conditioning is one of the most widely used paradigms to understand the underlying neural mechanisms of associative motor learning (for review, see Gerwig et al., 2007; Bracha et al., 2009; De Zeeuw and Ten Brinke, 2015). The contribution of the cerebellum has been studied in great detail (McCormick and Thompson, 1984). Fear conditioning, on the other hand, has been extensively used to study emotional learning, which relies centrally on the role of the amygdala (LeDoux, 2000; Phelps and LeDoux, 2005). Although studied in much less detail, the amygdala is also known to be involved in eyeblink conditioning. For example, inhibition of the amygdala slows down acquisition of conditioned eyeblink responses (Weisz et al., 1992; Neufeld and Mintz, 2001; Lee and Kim, 2004; Farley et al., 2016, 2018), whereas prior fear acquisition training facilitates the acquisition of conditioned eyeblinks (Neufeld and Mintz, 2001). The two-stage or two-factor theory of aversive conditioning is most commonly used to explain these observations (Konorski, 1967; Rescorla and Solomon, 1967; Thompson et al., 1987; Lennartz and Weinberger, 1992; Mintz and Wang-Ninio, 2001). In the first and fast stage, unspecific aversive responses, that is, conditioned fear responses, are thought to be acquired as an expression of learning and memory within the amygdala. In the second and slower stage, the specific aversive response, that is, the conditioned eyeblink response, is learned, which depends on associative plasticity within the cerebellum. Based on this two-stage theory of aversive conditioning, learned fear is a necessary prerequisite to acquire conditioned eyeblink responses, but is no longer needed after acquisition of the latter has occurred. In fact, a third stage of learning has been proposed, in which the initially acquired fear responses are extinguished. Magal and Mintz (2014) found that electrical activation of the cerebellar nuclei [mimicking conditioned response (CR) output] suppressed activation of the amygdala to an aversive periorbital electrical stimulation [mimicking the unconditioned stimulus (US)] in rats. The authors suggested that once conditioned eyeblinks are learned, the CR-related output of the cerebellar nuclei inhibits the US-related signal in the amygdala, leading to extinction of the amygdala-dependent conditioned fear responses. A comparable inhibitory feedback loop between the cerebellar nuclei and the inferior olive is known to contribute to the extinction of conditioned eyeblink responses in unpaired trials (Hesslow and Ivarsson, 1996; Medina et al., 2002; Bengtsson et al., 2007).

Recently, the two-stage (or three-stage) theory of learning has been challenged. Farley et al. (2016) found that inactivation of the amygdala impaired not only acquisition but also retention of conditioned eyeblink responses. Furthermore, amygdala-dependent modulation did not require memory consolidation in the amygdala (Steinmetz et al., 2017). Both observations are at variance with the two-stage theory. The authors proposed an alternative hypothesis, known as the sensory-gating hypothesis of the amygdala, with the amygdala gating inputs about the conditioned stimulus (CS) to the cerebellum. Gating of the CS is conceptualized as increasing selective attention to the CS, leading to increased salience of the CS and therefore increased cerebellar learning (Farley et al., 2016). The amygdala has known monosynaptic projections to the pontine nuclei (Mihailoff et al., 1989; Farley et al., 2016), and there is experimental evidence that the amygdala modulates eyeblink conditioning through these projections (Farley et al., 2018). More recent findings show that connections of the amygdala to the locus ceruleus and periaqueductal gray also play a role (Farley and Freeman, 2019).

Differentiation between these two assumptions has potential clinical relevance: in case the two-stage (or three-stage) theory is correct, eyeblink conditioning should, once the initial fast learning phase has been achieved, eventually suppress the amygdala. Conditioned eyeblinks, but possibly also other forms of cerebellar-dependent motor learning, may modify pathologic fear responses (Magal and Mintz, 2014). Although any clinical application would require that effects are longer lasting, and it is unclear whether the suppression is equally present in the presence of a US eliciting fear, this suppression may help to facilitate fear extinction, which is at the core of exposure therapy in the treatment of many anxiety disorders (for review, see Craske and Mystkowski, 2006; Craske et al., 2014). In case the sensory-gating hypothesis of the amygdala is correct, the amygdala continues to play a modulatory role in eyeblink conditioning once conditioned responses have been acquired. Therefore, conditioned eyeblinks should not alter or even impede the extinction of conditioned fear responses. We tested these two opposing possibilities in young and healthy human participants. On the first day, conditioned fear responses were acquired before the acquisition of conditioned eyeblink responses. On the second day, concomitant extinction of conditioned fear and conditioned eyeblink responses was performed.

## Materials and Methods

### Participants

Because menstrual cycle and oral contraceptives influence the acquisition and extinction of conditioned fear (Merz et al., 2018), only male participants were included in this study. The required sample size for the present study was calculated with G*Power software (Faul et al., 2009). One hundred participants divided into five groups were required to obtain a medium (*f *=* *0.25) effect size (Cohen, 1988), with a given significance level of α = 0.05, an assumed correlation among repeated measurements of *r* = 0.35, and a power (1 − β) of 0.9.

One hundred ten young and healthy male participants were recruited. Six participants had to be excluded from the analysis for technical reasons. Four participants did not finish the experiment. One hundred participants were included in the analysis (mean age, 23.58 ± 3.26 years; age range, 18–32 years). All participants were right handed according to the Edinburgh Handedness Inventory (Oldfield, 1971) with the group median [interquartile range (IQR)] score of 100 (82.95–100) on a point scale of −100 (pure left hander) to 100 (pure right hander). Only participants without any neurologic or psychiatric disorder were included. None were taking centrally acting drugs, and none were smoking more than 10 cigarettes per month. All participants had normal or corrected-to-normal vision and did not wear contact lenses regularly (which may impact eyeblink conditioning).

Each participant underwent neurologic examination before the start of the experiment, which was always unremarkable. Participants’ depression, anxiety, and stress levels were assessed using the DASS-21 (Depression, Anxiety and Stress Scale - 21 Items) questionnaire (Henry and Crawford, 2005; Norton, 2007). Scores were within the following normal ranges: depression score, median 2 (IQR, 0–4); anxiety score, 2 (IQR, 0–4.5), stress score, 6 (IQR, 2–10). Participants were asked to refrain from alcohol consumption 1 d before the experiment, and from caffeine 2 h before the experiment. The study was approved by the Ethics Committee of the University Hospital Essen and conforms to the principles laid down in the Declaration of Helsinki. All participants signed the written informed consent form. Participants were compensated for their participation with 60 €.

### Methods

**Figure 1.**
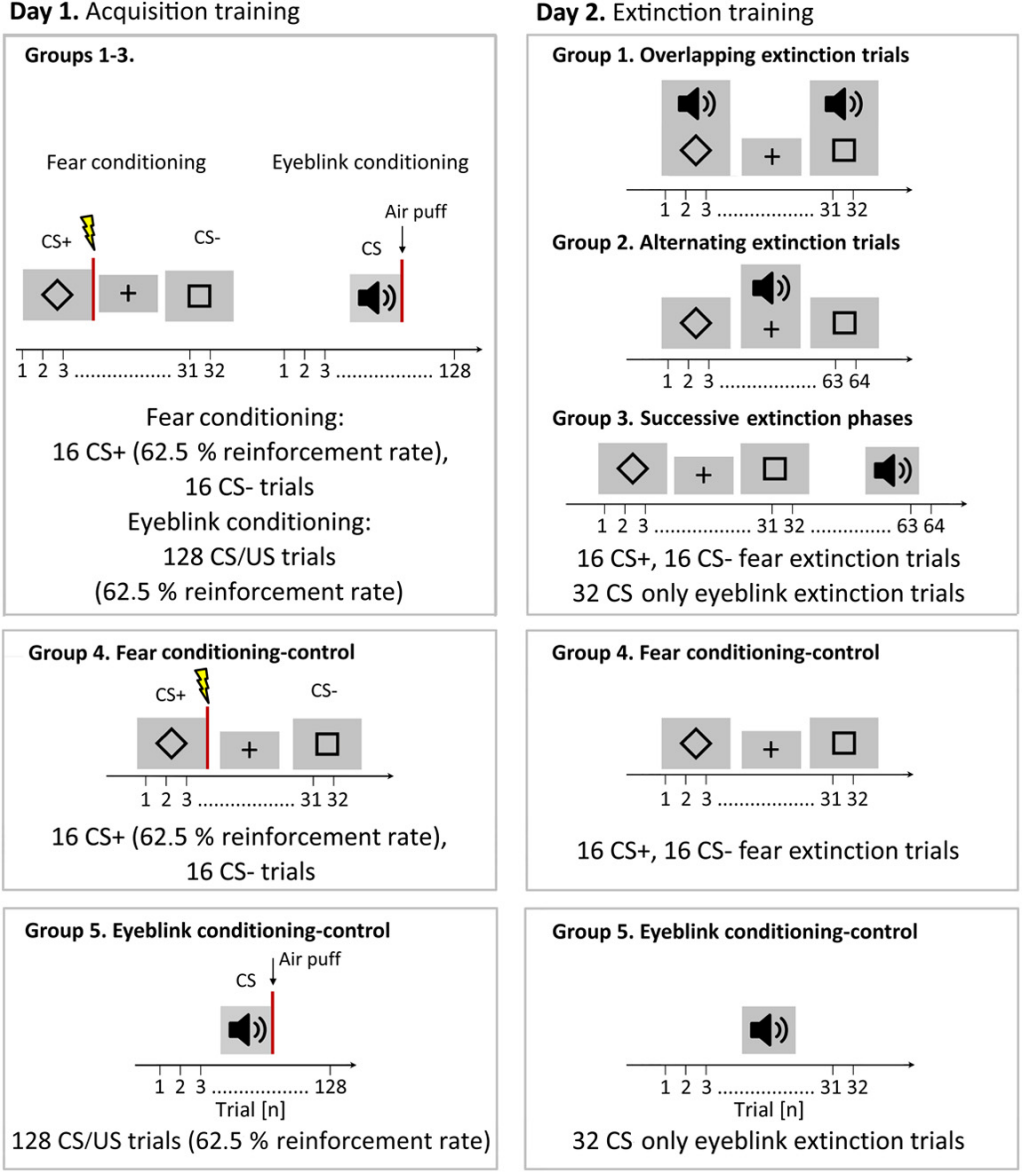
Summary of the experimental protocols in groups 1–5. Acquisition training protocols on day 1 are shown in the left column, and extinction training protocols on day 2 are shown in the right column. In fear conditioning, two geometrical figures (diamond and square) served as CSs, and electric shock (indicated by the flash symbol and red line) served as USs. In eyeblink conditioning, a tone (indicated by the speaker symbol) served as CS, an air puff as US (indicated by arrow and the red line). The number of trials is given on the *x*-axis. Between fear-conditioning trials, a fixation cross was shown. Further details can be found in the text.

Participants sat in a comfortable chair in front of a computer screen. The paradigms were implemented using the software Presentation (version 16.4; Neurobehavioral Systems). Participants were randomly assigned to one of five groups (20 participants/group; Fig. 1). The experiment was performed on 2 consecutive days. In Groups 1–3, fear acquisition training was performed followed by eyeblink acquisition on day 1. Partial reinforcement rates were used to slow down extinction learning (for review, see Lonsdorf et al., 2017). On day 2, extinction training took place. In group 1 (“overlapping extinction trials”), fear and eyeblink extinction trials were presented simultaneously. In group 2 (“alternating extinction trials”), individual fear and eyeblink extinction trials alternated. Based on the findings by Magal and Mintz (2014), the proposed modulatory effects on the amygdala were expected to be most prominent during the presentation of the eyeblink CS. Thus, effects on the extinction of learned fear responses were expected to be more prominent in group 1 (“overlapping extinction trials”) compared with group 2 (“alternating extinction trials”). Groups 3, 4, and 5 were control groups. In group 3 (“successive extinction phases”), fear extinction training was followed by eyeblink extinction. In group 4 (“fear conditioning-control”), fear acquisition training was performed on day 1, and fear extinction training on day 2. In group 5 (“eyeblink conditioning-control”), eyeblink acquisition training was performed on day 1, and eyeblink extinction on day 2.

Physiologic data [i.e., skin conductance responses (SCRs) and eyeblink electromyography (EMG)] responses, were recorded at a 1 kHz sampling rate with a modular measurement station with corresponding amplifier modules and propriety recording software (MP160, EDA100C, EMG100C, AcqKnowledge 5.0.2, BIOPAC Systems). Verbal reports of subjective experiences were used as additional outcome parameters (for review, see Lonsdorf et al., 2017).

### Fear conditioning

Differential fear conditioning was performed with two geometrical figures of identical brightness (diamond and square) serving as conditioned stimuli (CS^+^, CS^−^; Fig. 1; Merz et al., 2014; Utz et al., 2015). Geometrical figures were shown in black color against a gray background with a duration of 8 s. Between visual stimulus presentations, a black fixation cross on gray background was displayed [intertrial interval (ITI), 12 ± 1.2 s].

During fear acquisition training, 16 CS^+^ (62.5% reinforcement rate) and 16 CS^−^ trials were presented in a pseudorandomized order. During fear extinction training, a total of 32 (16 CS^+^ and 16 CS^−^) fear CS-only trials were presented. In group 1 (overlapping extinction trials), eyeblink CS-only onset was jittered and started 2144 ± 722 ms (range, between 943 and 3347 ms) after fear CS-only onset (ITI, 12 ± 1.2 s). In group 2 (alternating extinction trials), eyeblink CS-only trials alternated with fear CS-only trials (ITI, 15.1 ± 3.2 s). In group 3 (successive extinction phases), fear and eyeblink extinction training were performed as separate blocks, with the fear extinction training preceding eyeblink extinction training (interval between phases, 25 s; ITI between fear extinction trials, 18 ± 1.2 s; ITI between eyeblink extinction trials: 12 ± 1.2 s). A total of 32 eyeblink CS-only trials were presented in groups 1–3. In group 4 (fear extinction-control), only fear CS-only trials were presented (ITI, 18 ± 1.2 s).

A total of 16 CS^+^ and 16 CS^−^ trials were presented in each phase. An equal number of CS^+^ and CS^−^ trials were presented in the first and second half of fear acquisition and extinction training, respectively (fear acquisition training: 5 reinforced CS^+^ and three nonreinforced CS^+^ trials, 8 CS^−^ trials; extinction training: 8 CS^+^ trials, 8 CS^−^ trials). Use of the two geometrical figures as CS^+^ and CS^−^ was counterbalanced within each group. CS^+^ and CS^−^ were presented in a pseudorandom order that was the same for all participants. The following restrictions were applied: (1) each CS was presented no more than two consecutive times; (2) the first two and last acquisition trials were reinforced CS^+^ trials; and (3) the first two extinction trials were CS^+^ trials.

The US (100 ms duration) was composed of four consecutive 500 µs current pulses. The transcutaneous shock was applied to the back of the right hand via a concentric bipolar surface electrode (WASP Electrode, Specialty Developments) using a constant current stimulator (DS7A, Digitimer; maximum output voltage, 400 V). In reinforced trials, the US started 7.9 s after CS^+^ onset and coterminated with the CS^+^ (delay conditioning). The CS^−^ was never followed by a US. Before the experiment, electric shock intensity was individually adjusted. Intensity was increased until perceived as uncomfortable but not painful (mean current, 6.22  ± 3.56 mA). To counteract habituation to the US leading to weakening of the CRs (i.e., inhibition with reinforcement; Burns and Kimmel, 1975), 20% was added to the individual thresholds (mean added current, 1.24  ± 0.71 mA). Electric shock intensity was kept constant throughout the experiment. Participants, however, were allowed to ask for a decrease in intensity if needed. In two participants, intensity was decreased on request (by 10% and 15%, respectively) after the first presentation of the US. At the beginning of the experiment, participants were instructed that should they perceive a pattern between stimuli that this pattern would not change throughout the experiment.

**Figure 2.**
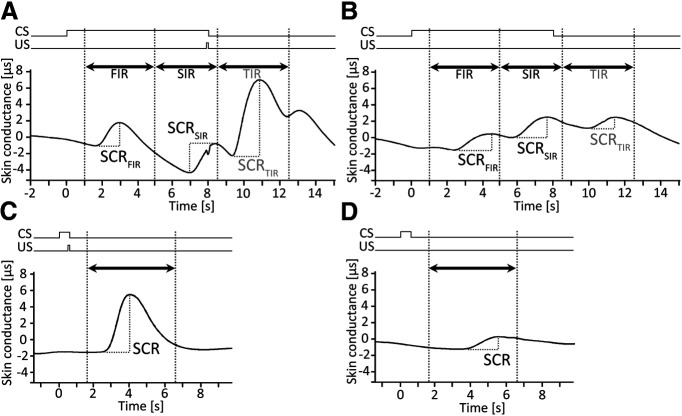
Examples of bandpass filtered individual SCRs. ***A***, ***B***, First, second, and third interval responses (SCR_FIR_, SCR_SIR_, SCR_TIR_) in a reinforced CS^+^ fear acquisition training trial (***A***) and an unreinforced CS^+^-only fear acquisition training trial (***B***). Time windows of the first (1.0–4.99 s after CS onset), second (5.0–8.49 s after CS onset), and third (8.5–13.0 s after CS onset) interval responses are indicated by arrows and hatched lines. ***C***, ***D***, SCRs in a paired CS/US eyeblink acquisition trial (***C***) and an unpaired CS-only eyeblink acquisition trial (***D***). SCR time window in eyeblink-conditioning trials (1–5 s after CS termination) is indicated by an arrow and hatched lines.

SCRs were obtained throughout the experiment using two SCR electrodes placed on the hypothenar of the left hand using electroconductive gel (GELLO Geltechnik). SCR data were bandpass filtered (0.5–10 Hz) to avoid high-frequency noise and low-frequency drifts. Raw SCRs were normalized through a logarithmic [ln(1 + SCR)] transformation. Processing of SCR data and semiautomated peak amplitude detection was performed using MATLAB software (releases 2017a and 2019a; MathWorks), followed by visual inspection and correction. SCRs were defined as the maximum trough-to-peak response amplitude within three predefined time windows (Prokasy and Ebel, 1967). Responses with an onset in the time window of 1.0–4.99 s after CS onset were defined as first interval response (SCR_FIR_), with an onset of 5.0-8.49 s after CS onset as second interval response (SCR_SIR_), and with an onset of 8.5–13.0 s after CS onset as third interval response (SCR_TIR_; Fig. 2*A*; Jentsch et al., 2020).

Normalized data and the distribution of residuals were tested for normality using the Shapiro–Wilk test. Since the normality test revealed a non-normal distribution of SCRs and the residuals (*p *<* *0.05), nonparametric statistical analysis was performed using the PROC Mixed procedure in SAS (SAS Studio 3.8, SAS Institute). SCRs were analyzed separately for fear acquisition training on day 1 and extinction training on day 2 using nonparametric ANOVA-type statistics for repeated measures (Brunner et al., 2002; Shah and Madden, 2004) with SCRs as dependent variable, group (groups 1–4) as a between-subject factor, and stimulus type (CS^+^ vs CS^−^) and trial (16 trials) as within-subject factors. *Post hoc* pairwise comparisons were performed where appropriate using least-squares means tests.

To display individual data points and effect sizes, Cumming estimation plots (Cumming, 2012) were generated using the web application of DABEST [“data analysis with bootstrap-coupled estimation” (http://www.estimationstats.com/); Ho et al., 2019].

### Eyeblink conditioning

A standard delay eyeblink-conditioning protocol was applied according to Gormezano and Kehoe (1975). The acquisition phase on day 1 consisted of 128 trials (80 paired CS/US trials with 48 CS-only trials interspersed; reinforcement rate, 62.5%). The extinction phase on day 2 consisted of 32 CS-only trials. A neutral tone (1 kHz; duration, 550 ms) was used as CS and was presented to the right ear via earplugs using an AD229 Diagnostic Audiometer (Interacoustics). Before the experiment, participants were tested for individual hearing thresholds. CS intensity was set to 80 dB. Ear defenders were used to reduce environmental noise. Participants wore an in house-built headband with an attached nozzle to apply an air puff as the US. The air puff (intensity, 400 kPa at source, 110 kPa at nozzle; duration, 100 ms) was directed to the outer canthus of the right eye with ∼1 cm distance to the skin. US onset was set 450 ms after CS onset. The US coterminated with the CS. The ITI was 14 ± 1.18 s. Participants watched a silent nature movie during the eyeblink acquisition phase. They were instructed that they would need to answer questions about the video after the experiment to maintain vigilance.

EMG electrodes were attached to the lower eyelids and nose bridge to obtain signals from the orbicularis oculi muscles. The collected data were preamplified, rectified, and filtered (bandpass filter frequency, between 100 Hz and 5 kHz; gain, 2000; sampling rate, 1 kHz). EMG signals were semiautomatically analyzed with a custom-made software (Zuchowski et al., 2014). In each trial, CR onset was defined as the time when EMG reached 7.5% of maximum amplitude. Trials were visually inspected. Recordings erroneously identified as CRs by the algorithm were manually corrected, that is, artifacts caused by technical errors (e.g., detachment of the electrode) or movement (e.g., talking of the participant). EMG responses occurring within 150 ms after CS onset were considered as reflexive (α) responses to the tone and not rated as CRs (Gerwig et al., 2005). In paired CS/US trials, responses occurring between 150 ms after CS onset and US onset were rated as CRs. In CS-only trials, responses occurring between 150 ms after CS onset and CS termination were rated as CRs. If spontaneous blinks occurred 550 ms before CS onset, responses were not counted as CRs. Blocks of eight trials (five paired CS/US trials and three unpaired CS-only trials in the acquisition phase; eight unpaired CS-only trials in the extinction phase) were used to calculate the percentage of CR incidence per block. Latencies of CR onset and peak time, CR area, and CR duration were also analyzed as detailed in Extended Data Figures 5-3, 5-4, 5-5, and 5-6.

Nonparametric ANOVA-type statistics for repeated measures were used for statistical analysis. Analyses were performed separately for the acquisition and extinction phases with CR incidence as the dependent variable, group (groups 1, 2, 3, and 5) as the between-subject factor, and block (16 in acquisition; 4 in extinction) as the within-subject factor. *Post hoc* comparisons were performed using least-squares means tests.

In addition, SCR data during eyeblink conditioning were analyzed. The maximum trough-to-peak-amplitude was calculated within a time window of 1–5 s following CS termination (Fig. 2*C*,*D*). During acquisition training, SCR data were analyzed separately for paired CS/US and unpaired CS-only trials, because SCR data are confounded by response to the US (air puff) in paired CS/US trials. Data of five consecutive CS/US trials and three consecutive CS-only trials were averaged, resulting in 16 CS/US and 16 CS-only blocks. During extinction training, SCR data were averaged across eight consecutive trials, resulting in four blocks. Nonparametric ANOVA-type statistics for repeated measures was calculated separately for paired and unpaired trials in acquisition training, and for unpaired trials in extinction training with SCR as the dependent variable, group (groups 1, 2, 3, 5) as the between-subject factor, and block (16 in acquisition; 4 in extinction) as the within-subject factor.

### Fear-conditioning questionnaires

Participants were required to answer questionnaires before and after fear acquisition training, and after fear extinction training. The questionnaires were print copies. Participants were required to rate valence and arousal to images of the CS^+^ and CS^−^ on a 9-point Likert scale to assess the affective components of learning (for review, see Lonsdorf et al., 2017). The scale went from “very pleasant” and “calm” to “very unpleasant” and “very excited,” respectively. The valence and arousal ratings were compared using nonparametric ANOVA-type statistics for repeated measures with the self-reported valence or arousal score as the dependent variable, group (groups 1, 2, 3, and 4) as the between-subject factor, and time (prior acquisition, postacquisition, post extinction) as the within-subject factor. Postacquisition training, participants were asked to rate US unpleasantness on a 9-point Likert scale from not unpleasant to very unpleasant, and to estimate the mean probability that a US occurred after presentation of the CS^+^ (i.e., CS/US contingency). Unpleasantness of the US was rated after fear acquisition training to control for possible effects of habituation and sensitization (for review, see Lonsdorf et al., 2017). Differences between groups were analyzed using nonparametric ANOVA-type statistics.

## Results

### Fear conditioning

#### Acquisition phase (day 1)

**Figure 3.**
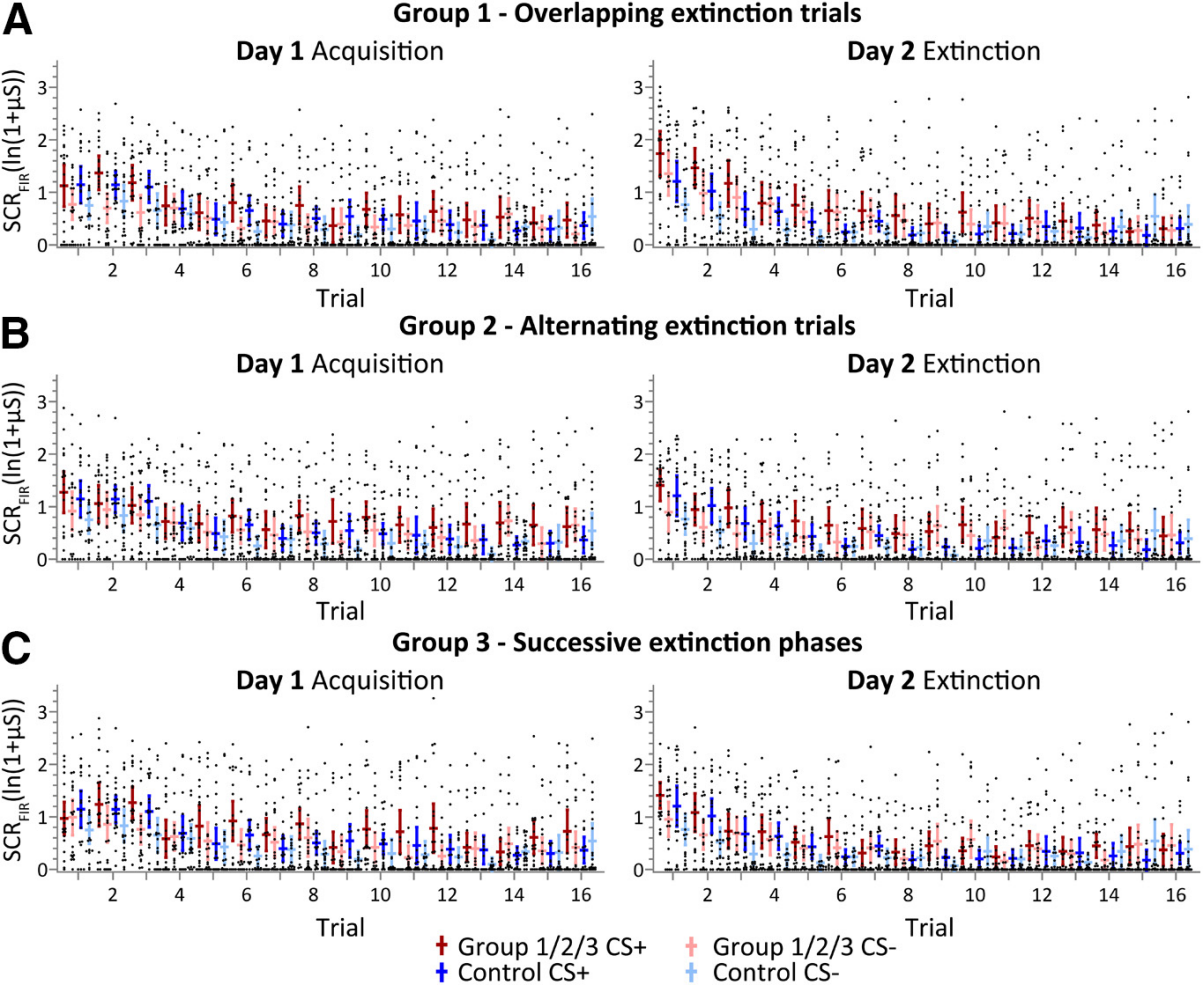
Fear-conditioning data. Group mean SCR_FIR_ and individual data on day 1 (fear acquisition training) and day 2 (extinction training) across trials. ***A–C***, Group 1 (overlapping extinction trials; shown in red) versus group 4 (control; shown in blue; ***A***); group 2 (alternating extinction trials; shown in red) versus group 4 (control; shown in blue; ***B***); and group 3 (successive extinction phases, shown in red) versus group 4 (control; shown in blue; ***C***). Horizontal lines represent group mean values; vertical lines indicate 95% confidence intervals. Black dots represent individual data points. See Extended Data Figure 3-1 for SCR_SIR_ data.

SCR_FIR_ values were significantly higher in CS^+^ trials compared with CS^−^ trials in all groups (Fig. 3*A–C*). Nonparametric ANOVA-type statistics revealed a significant main effect of trial (trials 1–16; *F*_(11.2)_ = 29.18, *p *<* *0.001), stimulus type (CS^+^ vs CS^−^; *F*_(1)_ = 24.91, *p *<* *0.001), and a significant trial × stimulus type interaction (*F*_(12.1)_ = 5.95, *p *<* *0.001). No significant main effects of group (*p *=* *0.64), group × stimulus (*p *=* *0.87), group × trial interaction (*p *=* *0.66), or group × stimulus × trial (*p *=* *0.86) were revealed. Similar findings were observed considering SCR_SIR_ (Extended Data Fig. 3-1). In CS^+^ trials, no significant group differences and interactions were observed regarding SCRs in the SCR_TIR_ (all *p* values > 0.42).

10.1523/ENEURO.0128-20.2020.f3-1 Figure 3-1 Fear conditioning data considering SCR_SIR_. Group mean SCR_SIR_ and individual data on day 1 (fear acquisition training) and day 2 (extinction training) across trials. (**A**) Group 1 (“overlapping extinction trials”; shown in red) vs. Group 4 (control; shown in blue), (**B**) Group 2 (“alternating extinction trials”; shown in red) vs. Group 4 (control; shown in blue), (**C**) Group 3 (“successive extinction phases”, shown in red) vs. Group 4 (control; shown in blue). Horizontal lines represent mean values, vertical lines indicate 95% confidence intervals. Black dots show individual data points. Fear acquisition training: SCR_SIR_ was significantly higher in CS+ trials compared to CS- trials in all groups (**A-C**). Non-parametric ANOVA-type statistics revealed a significant main effect of Trial (trial 1-16; F_12_ = 8.29, *p* < 0.001), Stimulus type (CS+ vs. CS-; F_1_ = 9.53, *p* = 0.0028) and a significant Trial x Stimulus type interaction (F_12.1_ = 6.24, *p* < 0.001). No significant main effect of Group (*p* = 0.8), and no significant Group x Stimulus (*p* = 0.24), Group x Trial (*p* = 0.82) or Group x Stimulus x Trial (*p* = 0.71) interactions were revealed. Fear extinction training: SCR_SIR_ were higher in CS+ trials compared to CS- trials in all groups at the beginning of extinction training phase. This difference disappeared in later trials (**A-C**). Non-parametric ANOVA-type statistics revealed a significant main effect of Trial (F_11.2_ = 13.70, *p* < 0.001), Stimulus type (F_1_ = 5.58, *p* = 0.0207) and a significant Trial x Stimulus type interaction (F_12_ = 2.17, *p* = 0.0112). The Group main effect (*p* = 0.49), and the Group x Stimulus (*p* = 0.18), Group x Trial (*p* = 0.14) and Group x Stimulus x Trial (*p* = 0.79) interactions were not significant. Download Figure 3-1, TIF file.

#### Extinction phase (day 2)

SCR_FIR_ values were higher in CS^+^ trials compared with CS^−^ trials in all groups at the beginning of extinction training. This difference disappeared in later trials (Fig. 3*A–C*). Nonparametric ANOVA-type statistics revealed a significant main effect of trial (trials 1–16; *F*_(9.73)_ = 30.91, *p *<* *0.001), stimulus type (CS^+^ vs CS^−^; *F*_(1)_ = 47.95, *p *<* *0.001), and a significant trial × stimulus type interaction (*F*_(11.5)_ = 1.93, *p *=* *0.0301). The group × trial interaction did not reach significance (*F*_(1)_ = 29.2, *p *=* *0.064). The group main effect (*p *=* *0.52), the group × stimulus interaction (*p *=* *0.75) and the group × stimulus × trial interaction (*p *=* *0.12) were not significant.

**Figure 4.**
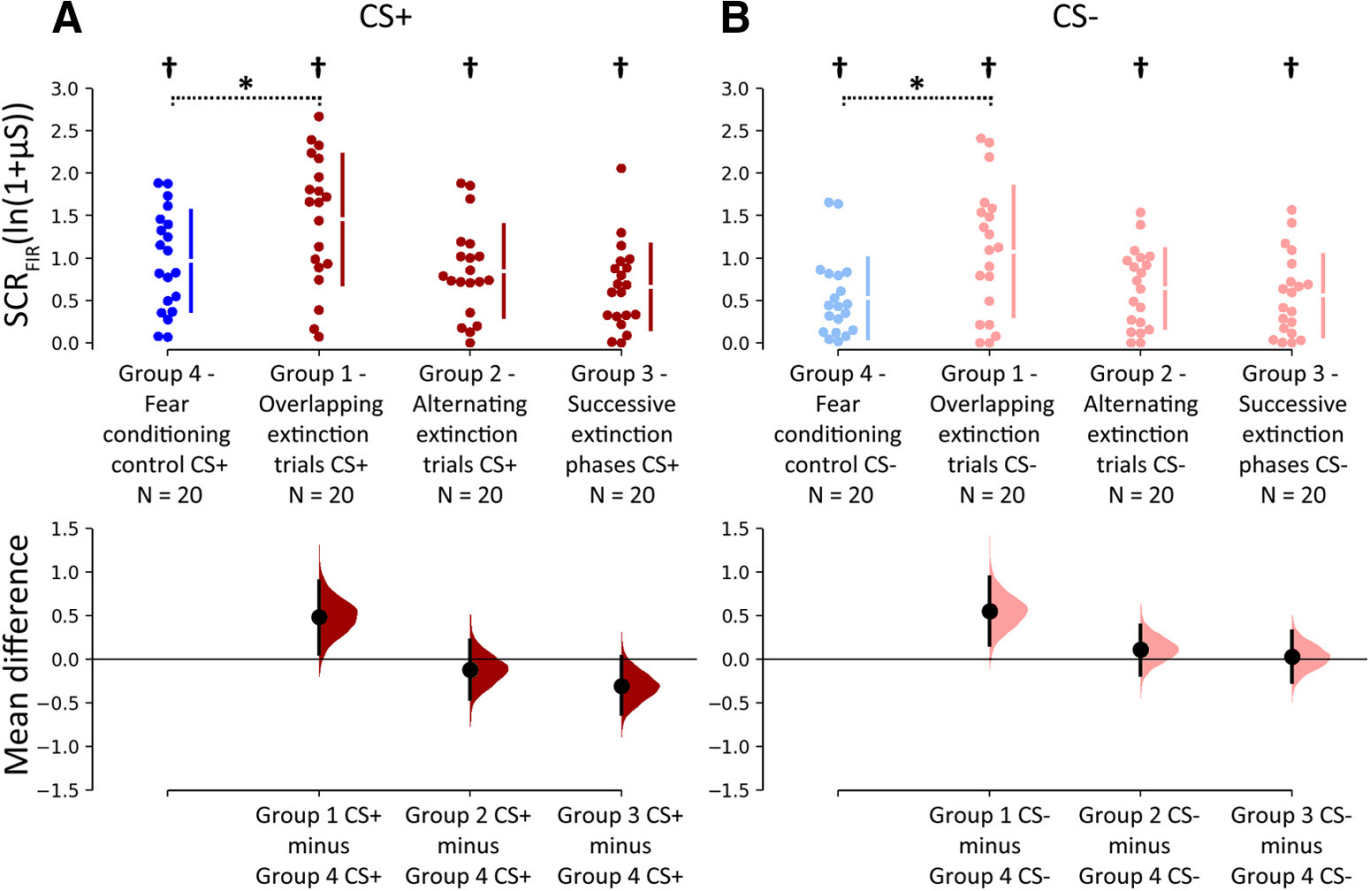
Recall of learned fear responses for SCR_FIR_ at the beginning of extinction training. ***A***, ***B***, Cumming estimation plots showing mean differences between groups 1 and 3 (shown in red) and the control group 4 (shown in blue) averaged across the first three extinction trials of CS^+^ (***A***) and CS^−^ (***B***) SCR_FIR_. Top, Dots represent individual data points. Gapped lines indicate group means (gap) and SDs. Bottom, Effect sizes. Black dots represent the mean differences between groups, and error bars indicate 95% CIs. 95% CIs are calculated by bootstrap resampling (Ho et al., 2019). Filled curves represent the bootstrap sampling distribution of the observed data. Dark colors, CS^+^; light colors, CS^−^. *Significant differences between respective stimuli between group 1 and group 4 (control; Mann–Whitney *U* tests, *p* values <0.05). †Significant SCR_FIR_ differences between CS^+^ and CS^−^ in the same group (Wilcoxon signed rank test, *p *<* *0.05). No group differences were observed considering SCR_SIR_ (Extended Data Fig. 4-1).

Closer inspection of Figure 3 showed that SCR_FIR_ values in the initial CS^+^ and CS^−^ trials, that is, during recall of learned fear responses, were higher in group 1 (overlapping extinction trials) compared with the control group 4, but not in group 2 (alternating extinction trials) and group 3 (successive extinction phases) compared with the control group 4. This difference is further illustrated in Figure 4 showing SCR_FIR_ averaged across the first three extinction trials. Mann–Whitney *U* tests revealed significant differences between groups 1 and 4 (CS^+^: *U* = 122, *z* = −2.110, *p *=* *0.035; CS^−^: *U* = 123, *z* = −2.083, *p *=* *0.037), but no significant differences comparing group 2 and group 4 (CS^+^: *U* = 177, *z* = −0.622, *p *=* *0.53; CS^−^: *U* = 157, *z* = −0.927, *p *=* *0.35), and group 3 and group 4 (CS^+^: *U* = 184, *z* = −0.433, *p *=* *0.67; CS^−^: *U* = 148, *z*  = −1.407, *p *=* *0.16). No group differences were observed considering SCR_SIR_ (Extended Data Figs. 3-1, 4-1).

10.1523/ENEURO.0128-20.2020.f4-1 Figure 4-1 Recall of learned fear responses at the beginning of the extinction phase. Cumming estimation plots showing mean differences between groups 1-3 (shown in red) and the control group 4 (shown in blue) averaged across the first three extinction trials of **A)** CS+ and **B)** CS- SCR_SIR_. Dots on upper panel represent individual data points. Gapped lines indicate group means (gap) and standard deviations. Lower panel shows effects sizes. Black dots represent mean differences between groups and error bars 95% confidence intervals (CI). 95% CI are calculated by bootstrap resampling (Ho et al., 2019). Filled curves represent the bootstrap sampling distribution of the observed data. Dark colors = CS+, light colors = CS. † indicates significant SCR_SIR_ differences between CS+ and CS- in the same group (Wilcoxon signed rank test, *p* < 0.05). Mann-Whitney U tests revealed no significant differences comparing Group 1 (CS+: U = 187, Z = -0.354, *p* = 0.72; CS-: U = 144, Z = -1.517, *p* = 0.13), Group 2 (CS+: U = 166, Z = -0.907, *p* = 0.37; CS-: U = 187, Z = -0.352, *p* = 0.73), and Group 3 (CS+: U = 143, Z = -1.542, *p* = 0.12; CS-: U = 148, Z = -1.407, *p* = 0.159) with Group 4, respectively. Download Figure 4-1, TIF file.

### Eyeblink conditioning

#### CR incidence

##### Acquisition phase (day 1)

**Figure 5.**
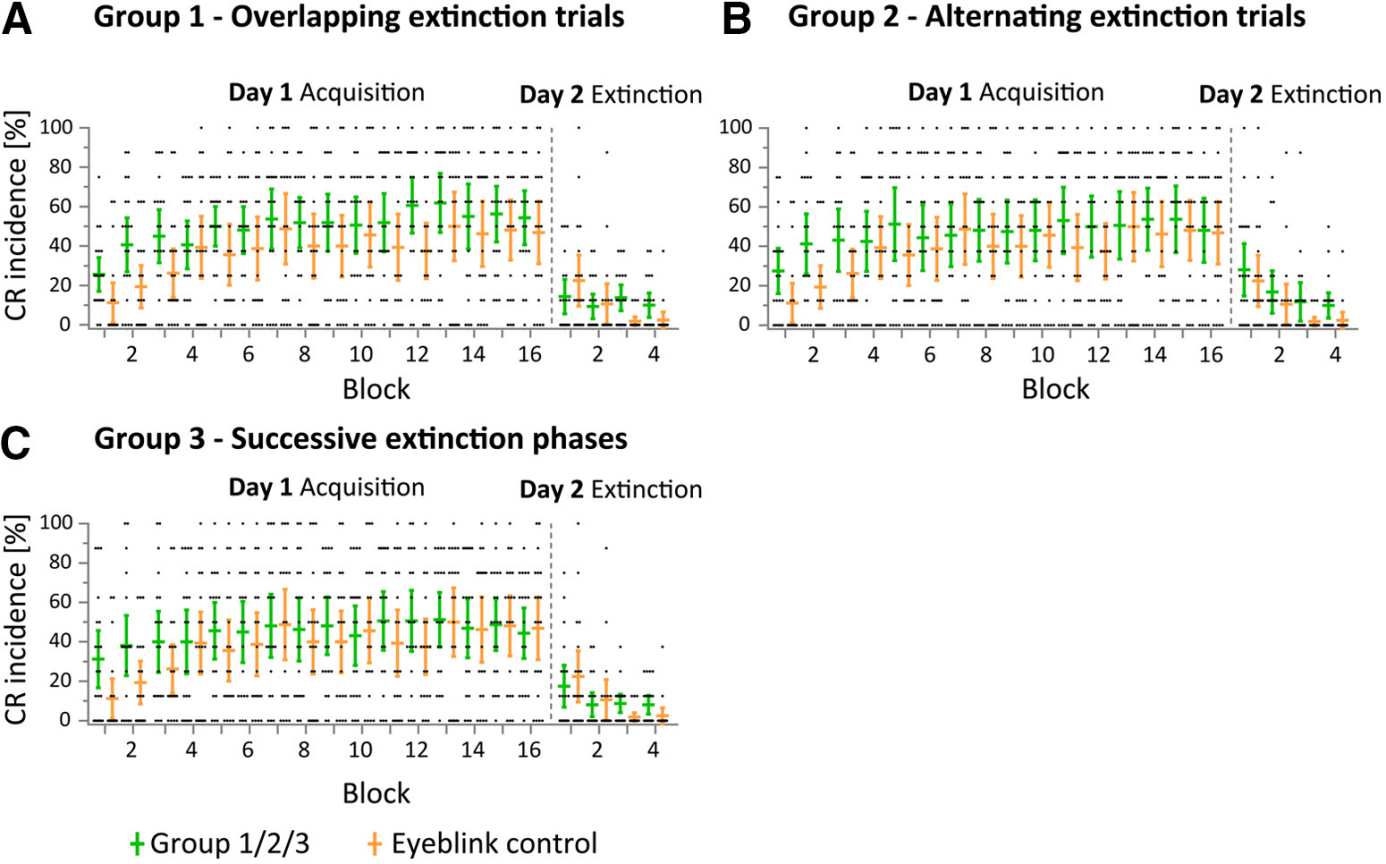
Eyeblink conditioning: group mean percentage CR incidences and individual data shown in the acquisition phase on day 1 (16 blocks and 8 trials), and in the extinction phase on day 2 (4 blocks and 8 trials). ***A–C***, Group 1 (overlapping extinction trials; shown in green) versus group 5 (control; shown in yellow; ***A***); group 2 (alternating extinction trials; shown in green) versus group 5 (control; shown in yellow; ***B***); and group 3 (successive extinction phases; shown in green) versus group 5 (control; shown in yellow; ***C***). Horizontal lines represent mean values, vertical lines indicate 95% confidence intervals. Black dots show individual data points. CR incidences for the first four acquisition blocks are shown in Extended Data Figure 5-1. Trial-by-trial analysis of the first acquisition and extinction blocks are shown in Extended Data Figure 5-2. Latencies of CR onset and peak time, and CR area and CR duration are presented in Extended Data Figures 5-3, 5-4, 5-5, and 5-6.

Figure 5 shows the mean percentage and 95% confidence intervals (CIs) of CR incidences across the 16 acquisition and 8 extinction blocks in the four groups. In the acquisition phase, a significant increase in CR incidence was observed in all groups. Taking all participants (*n *=* *80) together, the mean ± SD percentage of CR incidence increased from 23.91 ± 25.01% in the first acquisition block to 48.44 ± 31.1% in the last acquisition block. Nonparametric ANOVA-type statistics revealed a significant effect of block (*F*_(9.7)_ = 15.10; *p *<* *0.0001), but no group (*p *=* *0.59) or group × block interaction effects (*p *=* *0.45).

10.1523/ENEURO.0128-20.2020.f5-1 Figure 5-1. CR incidences shown for the first four acquisition blocks (block à eight trials). Cumming estimation plots showing mean differences between groups 1-3 (shown in green) and control group 5 (shown in yellow) for acquisition blocks **A)** 1, **B)** 2, **C)** 3 and **D)** 4. Dots on upper panel represent individual data points. Gapped lines indicate group means (gap) and standard deviations. Lower panel shows effects sizes. Black dots represent mean differences between groups and error bars 95% confidence intervals (CI). 95% CI are calculated by bootstrap resampling (Ho et al., 2019). Filled curves represent the bootstrap sampling distribution of the observed data. * indicates significant differences between respective stimuli between Groups 1/2/3 and Group 5 (least square means tests, *p* values < 0.05). Non-parametric ANOVA-type statistics performed in individual blocks revealed significant group differences in the first block only (Block 1: F_3_ = 3.87, *p* = 0.0124; Block 2: F_3_ = 2.51, *p* = 0.0646; Block 3: F_3_ = 1.49, *p* = 0.2551; Blocks 4-10 *p* > 0.41). *Post-hoc* pairwise comparisons revealed significantly increased CR incidences in Groups 1, 2 and 3 compared to the Group 5 (control) during the first acquisition block (least square means tests, all *p* values < 0.0086. Download Figure 5-1, TIF file.

10.1523/ENEURO.0128-20.2020.f5-2 Figure 5-2. Trial-by-trial analysis of CR incidences considering the first acquisition block and the first extinction block (blocks à eight trials). The presence of a CR in an individual trial in each participant was coded as 1, and the absence was coded as 0. (**A**) Group 1 (“overlapping extinction trials”); (**B**) Group 2 (“alternating extinction trials”); (**C**) Group 3 (“successive extinction phases”); (**D**) Group 5 (“Eyeblink conditioning-control”). Horizontal lines represent mean probability in each group that a CR occurred in a given trial, vertical lines indicate 95% confidence intervals. Colored dots show individual data points. Download Figure 5-2, TIF file.

10.1523/ENEURO.0128-20.2020.f5-3 Figure 5-3. Eyeblink conditioning: CR onset. CR onset latencies are expressed as time after CS onset. Grey shading represents the time of the US presentation in CS/US paired trials. Group mean CR onset latencies and individual data are shown in the acquisition phase on day 1 (16 blocks à 8 trials corresponding to 5 CS/US trials and 3 CS only trials per block), and in the extinction phase on day 2 (4 blocks à 8 trials). (**A**) Group 1 (“overlapping extinction trials”, shown in green) vs. Group 5 (control, shown in yellow); (**B**) Group 2 (“alternating extinction trials”, shown in green) vs. Group 5 (control, shown in yellow); (**C**) Group 3 (“successive extinction phases”, shown in green) vs. Group 5 (control, shown in yellow). Horizontal lines represent mean values, vertical lines indicate 95% confidence intervals. Black dots show individual data points. Acquisition phase: In paired CS/US trials, non-parametric ANOVA-type statistics revealed no significant effects of Block (*p* = 0.12), Group (*p* = 0.48) or Group x Block interactions (*p* = 0.58). In CS only trials, non-parametric ANOVA-type statistics revealed a significant effect of Block (F_11_ = 1.83; *p* = 0.047) - reflecting an earlier onset in later acquisition trials, that is a shift towards CS onset, a finding that is in accordance with the literature (e.g. Gruart et al., 2000; Koekkoek et al., 2002), but no Group (*p* = 0.36) or Group x Block interaction effects (*p* = 0.58). Extinction phase: Non-parametric ANOVA-type statistics revealed no significant main effect of Block (*p* = 0.49), Group (*p* = 0.72) or Group x Block interactions (*p* = 0.69). Download Figure 5-3, TIF file.

10.1523/ENEURO.0128-20.2020.f5-4 Figure 5-4. Eyeblink conditioning: CR peak time. Peak time latencies are expressed as time after CS onset. Grey shading represents the time of the US presentation in CS/US paired trials. In paired CS/US trials, CR peak time was defined at the time of maximum amplitude before US onset. In unpaired CS only trials, CR peak time was defined at the time of the maximum CR amplitude. Group mean CR peak time latencies and individual data are shown in the acquisition phase on day 1 (16 blocks à 8 trials corresponding to 5 CS/US trials and 3 CS only trials per block), and in the extinction phase on day 2 (4 blocks à 8 trials). (**A**) Group 1 (“overlapping extinction trials”, shown in green) vs. Group 5 (control, shown in yellow); (**B**) Group 2 (“alternating extinction trials”, shown in green) vs. Group 5 (control, shown in yellow); (**C**) Group 3 (“successive extinction phases”, shown in green) vs. Group 5 (control, shown in yellow). Horizontal lines represent mean values, vertical lines indicate 95% confidence intervals. Black dots show individual data points. Acquisition phase: Non-parametric ANOVA-type statistics revealed no significant main effect of Block (CS/US paired trials *p* = 0.27, CS only trials: *p* = 0.18), Group (CS/US paired trials *p* = 0.67, CS only trials: *p* = 0.57) or Group x Block interactions (CS/US paired trials *p* = 0.30, CS only trials: *p* = 0.87). Extinction phase: Considering CR peak time, non-parametric ANOVA-type statistics revealed no significant main effect of Block (*p* = 0.43), Group (*p* = 0.74) or Group x Block interactions (*p* = 0.68). Download Figure 5-4, TIF file.

10.1523/ENEURO.0128-20.2020.f5-5 Figure 5-5. Eyeblink conditioning: CR area under the rectified EMG curve (Arbitrary Units – AUs). Baseline CR area was assessed in an interval of 100 ms prior US onset in each trial. Baseline area (corresponding to the CR duration) was subtracted from the CR area. In paired CS/US paired trials, CR area was assessed in the time window between CR onset and (up to) US onset because of frequent overlap with the UR. In CS only trials, CR area was assessed from CR onset to CR termination. Group mean CR areas and individual data are shown in the acquisition phase on day 1 (16 blocks à 8 trials corresponding to 5 CS/US trials and 3 CS only trials per block), and in the extinction phase on day 2 (4 blocks à 8 trials). (**A**) Group 1 (“overlapping extinction trials”, shown in green) vs. Group 5 (control, shown in yellow); (**B**) Group 2 (“alternating extinction trials”, shown in green) vs. Group 5 (control, shown in yellow); (**C**) Group 3 (“successive extinction phases”, shown in green) vs. Group 5 (control, shown in yellow). Horizontal lines represent mean values, vertical lines indicate 95% confidence intervals. Black dots show individual data points.
Acquisition phase: In paired CS/US trials, non-parametric ANOVA-type statistics revealed no significant effects of Block (*p* = 0.42), Group (*p* = 0.0788) or Group x Block interactions (*p* = 0.91). In CS only trials, non-parametric ANOVA-type statistics revealed a significant effect of Group (F_3_ = 5.43; *p* = 0.0015), but no significant effect of Block (*p* = 0.23) or Group x Block (*p* = 0.36) interactions. *Post-hoc* pairwise comparisons revealed significantly increased CR area in CS only trials in Groups 1, 2 and 3 compared to the Group 5 (control) during the acquisition phase (least square means tests, all *p* values < 0.0231).
Extinction phase: Non-parametric ANOVA-type statistics revealed no significant main effect of Block (*p* = 0.18), Group (*p* = 0.14) or Group x Block interactions (*p* = 0.73). Download Figure 5-5, TIF file.

**Figure 6.**
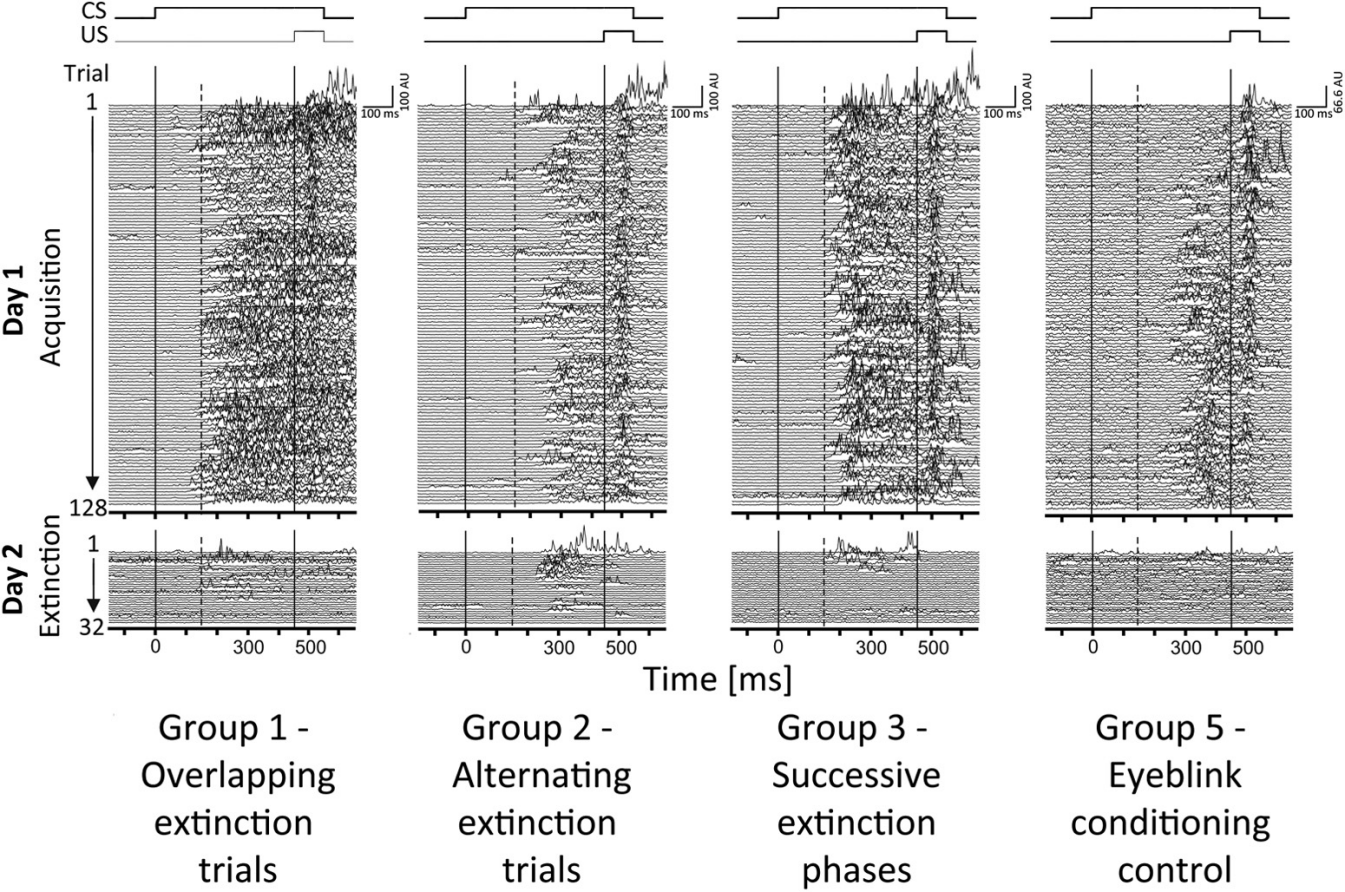
EMG eyeblink traces of individual participants from each group (groups 1, 2, 3, and 5). Rectified and filtered (100 Hz) EMG data of the orbicularis oculi muscle of 80 paired CS/US and 48 CS-only trials on day 1 (acquisition training) and 32 CS-only trials on day 2 (extinction training). The first (solid) vertical line indicates the beginning of the tone (CS), and the second (solid) vertical line indicates the beginning of the air puff (US). Responses occurring within the 150 ms interval after CS onset (dotted vertical line) were considered α blinks. AU, Arbitrary unit.

10.1523/ENEURO.0128-20.2020.f5-6 Figure 5-6. Eyeblink conditioning: CR duration. In paired CS/US paired trials, CR duration was assessed in the time window between CR onset and (up to) US onset because of overlap with the UR. In CS only trials, CR duration was assessed from CR onset to CR termination. Group mean CR durations and individual data are shown in the acquisition phase on day 1 (16 blocks à 8 trials corresponding to 5 CS/US trials and 3 CS only trials per block), and in the extinction phase on day 2 (4 blocks à 8 trials). (**A**) Group 1 (“overlapping extinction trials”, shown in green) vs. Group 5 (control, shown in yellow); (**B**) Group 2 (“alternating extinction trials”, shown in green) vs. Group 5 (control, shown in yellow); (**C**) Group 3 (“successive extinction phases”, shown in green) vs. Group 5 (control, shown in yellow). Horizontal lines represent mean values, vertical lines indicate 95% confidence intervals. Black dots show individual data points. * indicates significant differences between respective stimuli between Groups 1/2/3 and Group 5 (least square means tests, *p* values <0.05). Acquisition phase: In paired CS/US trials, non-parametric ANOVA-type statistics revealed no significant effects of Block (*p* = 0.28), Group (*p* = 0.44) or Group x Block interactions (*p* = 0.73). In CS only trials, non-parametric ANOVA-type statistics revealed a significant effect of Group (F_3_ = 8.09; *p* = 0.0002) and Group x Block interactions (F_28.1_ = 2; *p* = 0.0167), but no Block (*p* = 0.0708) effect. *Post-hoc* pairwise comparisons revealed significantly increased CR duration in CS only trials in Groups 1 and 2 compared to Group 5 (control) during the acquisition phase (least square means tests, all *p* values < 0.0055) but not Group 3 (*p* = 0.09). Extinction phase: Non-parametric ANOVA-type statistics revealed a significant main effect of Group (F_2.79_ = 3.89; *p* = 0.0157) but no Block (*p* = 0.49) or Group x Block interactions (*p* = 0.16). *Post-hoc* pairwise comparisons revealed significantly increased CR duration in Group 1 compared to Group 5 (control) during the extinction phase (least square means tests, *p* = 0.0225) but not for Groups 2 and 3 (*p* > 0.06). Download Figure 5-6, TIF file.

Closer inspection of Figure 5 revealed that CR incidence was higher in the three groups that had received prior fear conditioning (groups 1–3, indicated in green) compared with the control group (group 5, indicated in yellow). This difference was most marked in the first three acquisition blocks. Nonparametric ANOVA-type statistics performed in individual blocks revealed significant group differences in the first block only (block 1: *F*_(3)_ = 3.87, *p *=* *0.0124; block 2: *F*_(3)_ = 2.51, *p *=* *0.065; block 3: *F*_(3)_ = 1.49, *p *=* *0.26; blocks 4–10, *p *>* *0.41). *Post hoc* pairwise comparisons revealed significantly increased CR incidence in groups 1, 2, and 3 compared with that in group 5 (control) during the first acquisition block (least-squares means tests, all *p* values < 0.0086; Extended Data Fig. 5-1). The difference between control and experimental groups is further illustrated in Extended Data Figure 5-2, showing a trial-by-trial analysis of the first acquisition block, and in Figure 6, showing EMG eyeblink traces of individual participants from each group.

Closer inspection of EMG eyeblink traces in Figure 6 showed that responses occurred earlier in the three groups having received prior fear learning, compared with control participants, and responses sustained until US onset. CR onset and peak time latencies showed a high degree of variability within individual participants and were not significantly different between groups (group effects, group × block interaction effects: all *p* values > 0.48; Extended Data Figs. 5-3, 5-4). In CS-only trials, nonparametric ANOVA-type statistics with CR duration as the dependent variable revealed significant effects of group and group × block interaction (all *p* values <0.0167); the block effect was not significant (*p *=* *0.0708). In CS/US trials, no significant main effects of block, group or a group × block interaction were revealed (all *p* values >0.28; Extended Data Fig. 5-5). CR area was also significantly different between groups in CS-only trials (*p *=* *0.0015), but not in CS/US trials (*p *=* *0.0788); main effects of block and group × block interaction were not significant (all *p* values >0.23; Extended Data Fig. 5-6).

##### Extinction phase (day 2)

In the extinction phase, CR incidence decreased significantly in all groups (Fig. 5). Considering all participants together, the mean CR incidence decreased from 20.63 ± 24.77% in the first extinction block to 7.65 ± 11.85% in the last extinction block. During late extinction (extinction blocks 3–4), mean CR incidences were higher in groups 1–3 (Fig. 5, indicated in green) compared with group 5 (Fig. 5, control, indicated in yellow). Nonparametric ANOVA-type statistics revealed a significant main effect of block (*F*_(2.73)_ = 7.42; *p *<* *0.0002). No significant main effect of group or group × block interaction were revealed (*p *>* *0.08).

**Figure 7.**
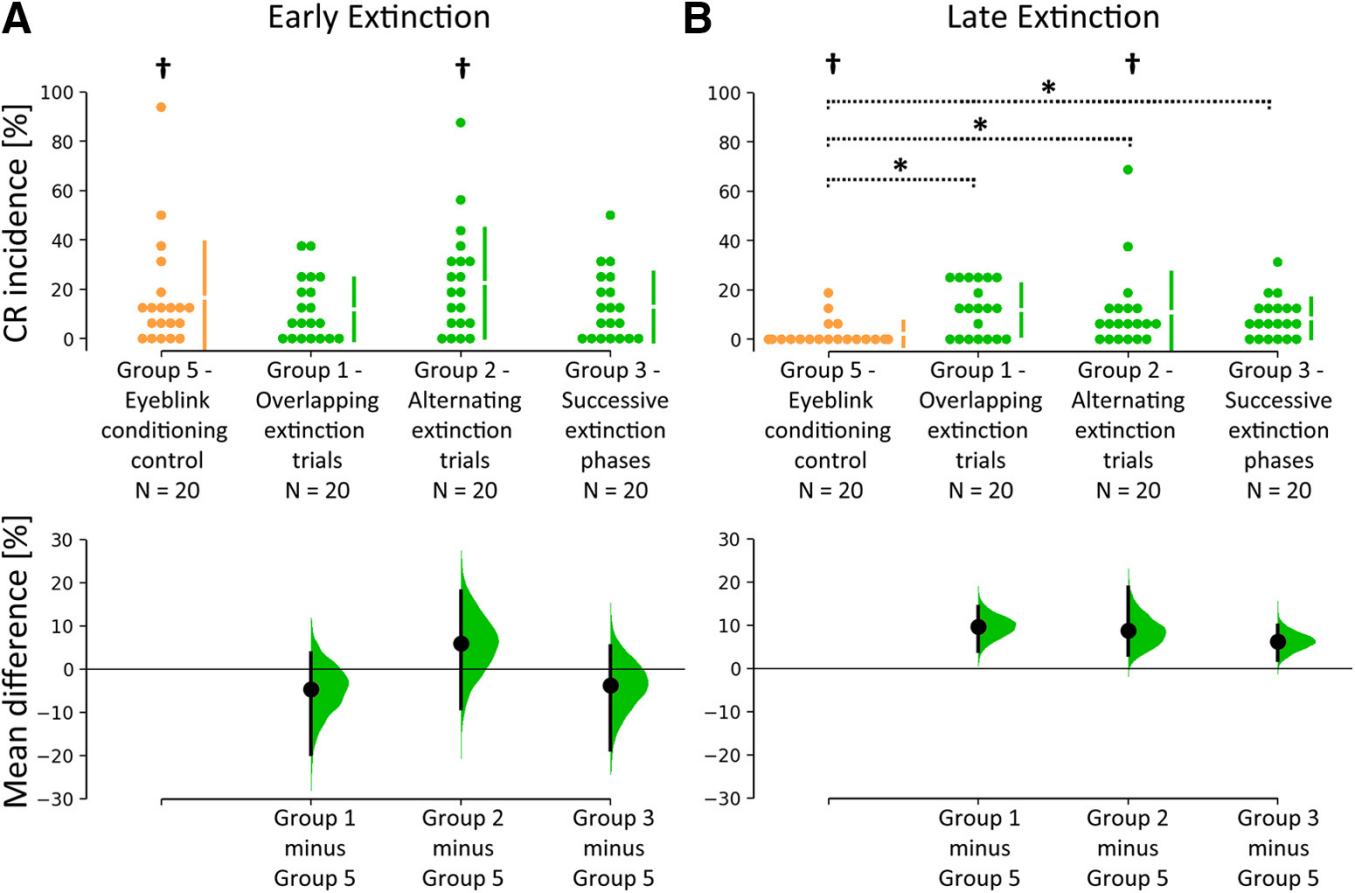
CR incidences in early and late extinction. ***A***, ***B***, Cumming estimation plot showing mean differences between groups 1 and 3 (shown in green) and control group 5 (shown in yellow) for early (***A***) and late (***B***) extinction (blocks in 16 trials). Top, Dots represent individual data points. Gapped lines indicate group means (gap) and SDs. Bottom, Effect sizes. Black dots represent mean differences between groups, and error bars indicate 95% CIs. 95% CI are calculated by bootstrap resampling (Ho et al., 2019). Filled curves represent the bootstrap sampling distribution of the observed data. *Significant differences between respective stimuli between Groups 1, 2, and 3 and group 5 (least squares means tests, *p* values <0.05). †Significant CR differences between early and late extinction in the same group (least square means tests, *p *<* *0.05).

*Post hoc* nonparametric ANOVA-type statistics comparing CR incidence in early extinction (trials 1–16) with late extinction (trials 17–32) revealed a significant main effect of block (early vs late; *F*_(1)_ = 12.53; *p *=* *0.0007), and a significant group × block interaction (*F*_(3)_ = 3.76; *p *= 0.0143). The group effect was not significant (*p *=* *0.12). *Post hoc* pairwise comparisons comparing early and late extinction revealed a significant CR reduction in group 2 (22.5 ± 22.16% to 10.94 ± 16.21%; least squares means test, *p *=* *0.0139) and the control group 5 (16.56 ± 22.51% to 2.19 ± 5.08%; least squares means test, *p *=* *0.0001), but not in groups 1 and 3 (least squares means tests, *p* values >0.45). *Post hoc* pairwise comparisons between groups revealed higher mean CR incidence in late extinction in groups 1, 2, and 3 (11.88 ± 12.65%, 22.5 ± 22.16%, and 12.81 ± 13.97%, respectively) compared with control group 5 (2.19 ± 5.08%, least squares means tests, all *p* values <0.0063). No significant differences were found in early extinction (least squares means tests, all *p* values >0.28; Fig. 7).

In the extinction phase, CR onset and peak time latencies, and CR area were not significantly different between groups (group effects, group × block interaction effects: all *p* values >0.14; Extended Data Figs. 5-3, 5-4, 5-6). Nonparametric ANOVA-type statistics considering CR duration revealed a significant main effect of group (*F*_(2.79)_ = 3.89; *p *=* *0.0157); the effects of block and group × block interaction were not significant (all *p* values >0.16; Extended Data Fig. 5-5).

#### Skin conductance responses

##### Acquisition phase (day 1)

**Figure 8.**
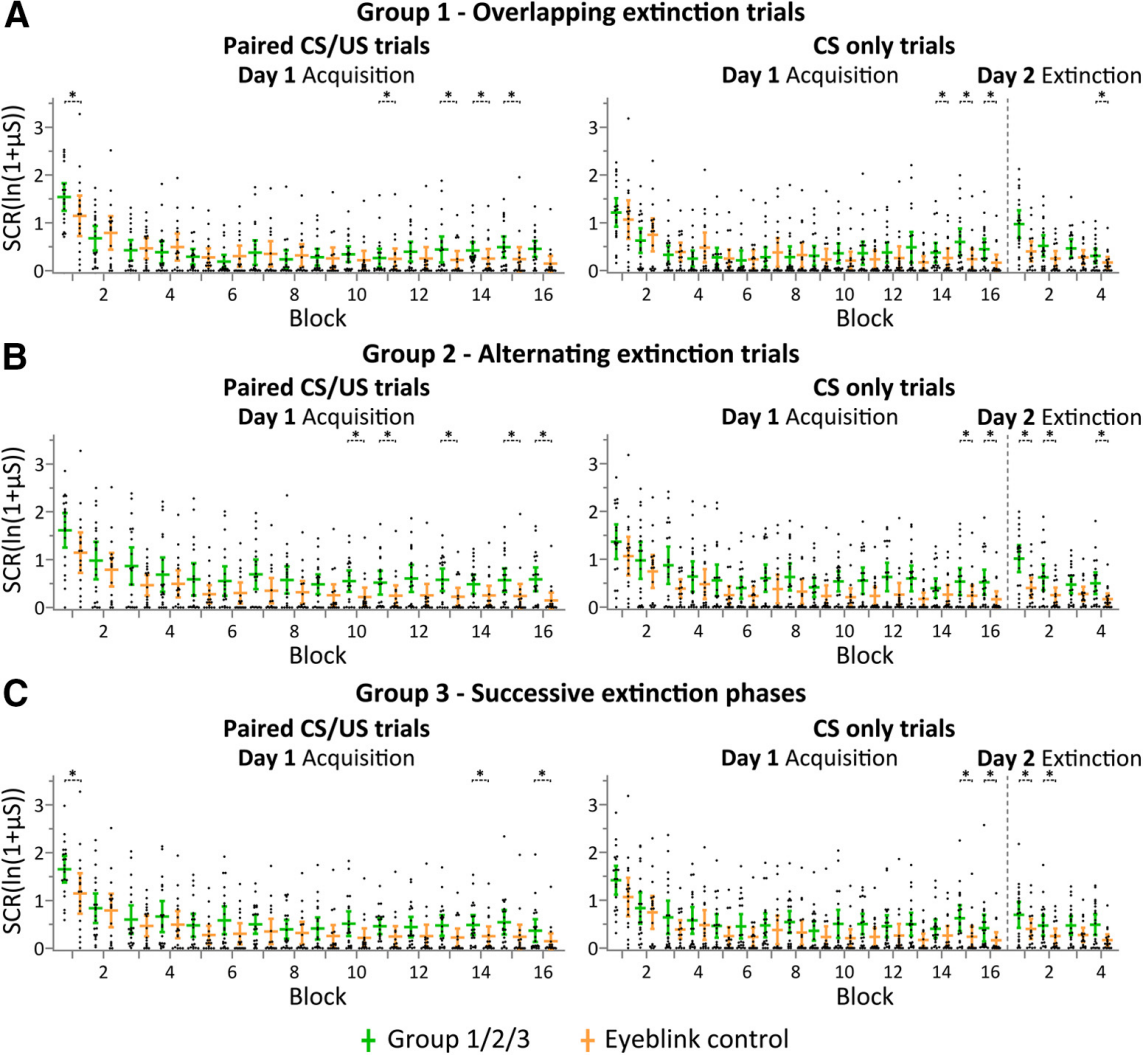
Eyeblink conditioning: group mean SCRs per block and individual data during eyeblink acquisition training in paired CS/US trials (5 trials/block), during eyeblink acquisition training in CS-only trials (3 trials/block), and during eyeblink extinction training in CS-only trials (8 trials/block). ***A–C***, Group 1 (overlapping extinction trials; shown in green) versus group 5 (control; shown in yellow; ***A***); group 2 (alternating extinction trials; shown in green) versus group 5 (control; shown in yellow; ***B***); and group 3 (successive extinction phases; shown in green) versus group 5 (control; shown in yellow; ***C***). Horizontal lines represent mean values, and vertical lines indicate 95% CIs. Black dots show individual data points. *Significant differences between respective stimuli between groups 1, 2, and 3 and group 5 (least squares means tests, *p* values < 0.05).

As expected, SCR analysis revealed higher SCR peak amplitudes in CS/US paired trials compared with CS-only trials during early acquisition blocks in all groups (Fig. 8). This difference decreased in later blocks. In CS/US paired and CS-only trials, SCR peak amplitudes tended to be higher in groups 1–3 compared with group 5 (Fig. 8, control, indicated in yellow). This difference was most prominent at the end of fear acquisition training. Considering paired CS/US trials, nonparametric ANOVA-type statistics revealed a significant main effect of block (*F*_(8.23)_ = 40.52, *p *<* *0.0001) and block × group interaction (*F*_(24.6)_ = 1.79, *p *=* *0.0114). Considering CS-only trials, nonparametric ANOVA-type statistics revealed a significant main effect of block (*F*_(10.8)_ = 16.0, *p *<* *0.0001) and a significant block × group interaction (*F*_(32.4)_ = 1.63, *p *=* *0.0156). No significant main effects of group (paired: *p *=* *0.15; unpaired: *p *=* *0.25) were revealed. *Post hoc* comparisons revealed significantly higher SCR peak amplitudes in group 1, 2, and 3 compared with the control group considering the following acquisition blocks: paired trials: group 1, blocks 1, 11, and 13–15; group 2, blocks 10, 11, 13, 15, and 16; group 3, blocks 1 and 14–16; unpaired trials: group 1, blocks 14–16; groups 2 and 3, blocks 15 and 16 (least squares means tests, all *p* values <0.0477).

##### Extinction phase (day 2)

SCRs during extinction training were significantly higher in groups 1, 2, and 3 compared with the control group 5 (Fig. 8). This difference was most prominent in the first extinction block. Nonparametric ANOVA-type statistics revealed a significant main effect of block (*F*_(2.56)_ = 38.31, *p *<* *0.0001) and group (*F*_(2.99)_ = 2.78, *p *=* *0.0475), and a significant block × group interaction (*F*_(7.65)_ = 2.56, *p *=* *0.0127). *Post hoc* comparisons revealed significantly higher SCRs comparing groups 2 and 3 with the control group 5 (least squares means tests, all *p* values <0.046) and a close to significant difference comparing groups 1 and 5 (least squares means test, *p *=* *0.0511). Pairwise group × block comparisons of groups 1, 2, and 3 with the control group 5 revealed significantly higher SCRs in the following blocks: group 1 versus 5, block 4; group 2 versus 5, blocks 1, 2, 4; group 3 versus 5, blocks 1, 2 (least squares means tests, all *p* values <0.049).

#### Fear conditioning questionnaires

##### Valence

Prior acquisition training and valence ratings of the CS^+^ and CS^−^ were not significantly different from each other (Table 1). Post-acquisition training, valence of the CS^+^ was rated as less pleasant compared with the CS^−^. A small difference in valence ratings remained post-extinction training (a finding frequently seen in the literature; e.g., Ernst et al., 2019). There was no difference between groups [group 1, 2, 3, and group 4 (control)]. Nonparametric ANOVA-type statistics revealed a significant main effect of time (prior acquisition vs post-acquisition vs post-extinction training: *F*_(1.84)_ = 4.03, *p *=* *0.023), stimulus (CS^+^ vs CS^−^: *F*_(1)_ = 81.56, *p *<* *0.0001), and a stimulus × time interaction (*F*_(1.91)_ = 48.68, *p *<* *0.0001). The group main effect (*p *=* *0.9), the group × stimulus interaction (*p *=* *0.9), and the group × stimulus × time interaction (*p *=* *0.35) were not significant. *Post hoc* tests showed significantly less pleasant valence rating toward CS^+^ than CS^−^ post-acquisition and post-extinction training (least squares means tests, all *p* values <0.0002), but not prior acquisition training (least squares means test, *p *=* *0.6). Valence ratings toward the CS^+^ post-acquisition training were rated significantly less pleasant compared with prior acquisition training, with the CS^−^ ratings showing the opposite effect (least square means tests, all *p* values <0.0001).

**Table 1:** Fear-conditioning questionnaires

Time of assessment	Group 1—overlapping extinction trials		Group 2—alternating extinction trials		Group 3—successive extinction phases		Group 4—fear conditioning control	
	CS^+^	CS^−^	CS^+^	CS^−^	CS^+^	CS^−^	CS^+^	CS^−^
Valence ratings (1, uncomfortable; 9, comfortable)								
Prior acquisition	5 (5–6)	5 (5–7)	5.5 (5–7)	5.5 (5–7)	5 (5–6.25)	5 (5–7)	5 (5–7)	5 (5–7)
Post-acquisition training	**3 (2.75**–5)*†	**7 (6.75**–8.25)*†	**3.5 (2**–5)*†	**7.5 (6**–9)*†	**4 (3**–5)*†	**7 (6**–8.25)*†	**4 (3**–5)*†	**7 (6**–8.25)*†
Post-extinction training	**5 (3**–7)^*^	**7 (5.75**–7.25)*†	**5 (5**–6.25)^*^	**7 (5**–8)*†	**5 (5**–7)^*^	**7 (5**–8)*†	**5 (5**–7)^*^	**6.5 (5**–8)*†
Arousal ratings (1, calm; 9, excited)								
Prior acquisition	3 (1–5)	2.5 (1–5)	3.5 (2–5)	3 (2–5)	3 (1–5)	2.5 (1–5)	3.5 (2–5)	5 (1.75–5)
Post-acquisition training	**5.5 (3.75**–7)*†	**2 (1**–3)*†	**5 (4.5**–7)*†	**1.5 (1**–3)*†	**6 (3**–7)*†	**1 (1**–3.25)*†	**5 (4.5**–7)*†	**2 (1**–4.25)*†
Post-extinction training	**3.5 (2.75**–6)^*^	**3 (1.75**–3.25)*†	**4 (2**–5)^*^	**2 (1**–3)*†	**3 (1**–5)^*^	**2 (1**–5)*†	**4 (2**–5)^*^	**2.5 (1**–5)*†

Self-reported median (IQR) valence and arousal ratings prior acquisition training, post-acquisition training and post-extinction training. Statistical significances (*p *<* *0.5) are indicated in bold.

* Significant differences between pre-acquisition training and post-acquisition training.

† Significant differences between CS^+^ and CS^−^ (least squares means tests, *p *<* *0.05).

##### Arousal

Prior acquisition training, arousal ratings of the CS^+^ and CS^−^ were not different from each other (Table 1). Post-acquisition, arousal toward the CS^+^ was rated higher compared with the CS^−^. A small difference in valence ratings remained post-extinction. There was no difference between groups [group 1, 2, and 3, and group 4 (control)]. Nonparametric ANOVA-type statistics revealed a significant main effect of time (prior acquisition vs post-acquisition- vs post-extinction training; *F*_(1.71)_ = 5.33, *p *=* *0.009), Stimulus (CS^+^ vs CS^−^; *F*_(1)_ = 56.72, *p *<* *0.0001) and stimulus × time interaction (*F*_(1.94)_ = 33.34, *p *<* *0.0001). The group main effect (*p *=* *0.49), the group × stimulus interaction (*p *=* *0.86), and group × stimulus × time interactions (*p *=* *0.94) were not significant. *Post hoc* tests showed significantly higher arousal ratings toward CS^+^ than CS^−^ post-acquisition training and post-extinction training (least squares means tests, all *p* values <0.0001), but not prior acquisition training (least square means test, *p *=* *0.55). Arousal toward the CS^+^ post-acquisition training was rated significantly higher compared with prior acquisition training, whereas arousal toward CS^−^ was rated significantly less compared with prior acquisition training (least square means tests, all *p* values <0.0004).

##### US unpleasantness and CS–US contingency

Post-acquisition training, the median US unpleasantness rating was 7 (IQR, 6–8) on a Likert scale from 1 (not unpleasant) to 9 (very unpleasant) considering all participants. There was no significant difference between groups (nonparametric ANOVA-type statistic, *p *=* *0.13). Post-acquisition training, participants reported that they recognized a pattern between CS^+^ and US contingency after 2.9 ± 1.4 min. Across all participants, the mean probability that a US occurred after presentation of the CS^+^ was estimated as 63.9 ± 17.8%, and after presentation of the CS^−^ as 2.53 ± 7.8% [0% probability by 68 of 80 (85%) participants]. There was no significant difference between groups (nonparametric ANOVA-type statistic, *p *=* *0.39).

## Discussion

The concomitant presentation of eyeblink- and fear-conditioning stimuli did not facilitate extinction learning but facilitated recall of previously learned fear responses. Furthermore, the extinction of conditioned eyeblink responses was impeded and was accompanied by increased autonomic fear responses. Findings do not support the hypothesis that conditioned eyeblink responses, once established, suppress conditioned fear responses (Magal and Mintz, 2014). The possible interactions between eyeblink and fear conditioning are discussed in more detail below.

The amygdala is critically involved in the acquisition and retention of learned fear responses (Weisz et al., 1992; Rogan et al., 1997; Rosenkranz et al., 2003; Schroeder and Shinnick-Gallagher, 2005; Butler et al., 2018). The extinction of learned fear requires inhibition of the amygdala (Amano et al., 2010; Herry et al., 2010; Krabbe et al., 2018). Thus, in case the assumption is correct that conditioned eyeblink responses lead to inhibition of the amygdala (Magal and Mintz, 2014), concomitant conditioned eyeblinks should reduce recall and accelerate extinction of conditioned fear responses. This was not the case. The concomitant presentation of conditioned eyeblink and fear stimuli had no significant impact on fear extinction learning. However, overlapping CSs resulted in increased recall of conditioned fear responses. The increase was most marked for SCRs in the FIR window (1–4.99 s following CS onset). This agrees with findings that conditioning-related changes of SCR amplitudes are most prominent in the first 3–4 s following CS onset (Pineles et al., 2009; Jentsch et al., 2020). It has been argued that FIRs reflect orienting or novelty responses (Ohman, 1972, 1974). Other studies suggest that FIRs are also related to associative processes, in particular during extinction (Jentsch et al., 2020). Increased recall of learned fear responses is in good agreement with the phenomenon of additive response summation: following individual conditioning for two different CSs, the compound presentation of the two CSs results in a CR that is the sum of the responses to each of the individual CSs (Hull, 1943; Kimble, 1961; Wolf, 1963; Weiss, 1964, 1972; Miller, 1969). The response increase is most obvious when CSs from different modalities are used to learn the same response (Pérez et al., 2018). In the present study, a visual fear CS and a tone eyeblink CS were used. Both result in conditioned fear responses. Additive response summation suggests that fear responses accompanying initial eyeblink conditioning are not fully suppressed after the specific aversive motor response has been developed. In fact, Lindquist et al. (2010) also provided evidence that fear conditioning in eyeblink conditioning is an autonomous learning process that is not turned off when conditioned eyeblink responses have been acquired. They found that preceding eyeblink conditioning resulted in facilitated acquisition of conditioned fear and increased conditioned fear responses.

As expected, prior fear conditioning accelerated the acquisition of conditioned eyeblink responses, accompanied by increased autonomic fear responses. Findings were most obvious in the first conditioning block. This agrees with the observation that learning occurs mainly in the first block of 10 conditioning trials in humans (Kjell et al., 2018). Previous findings on accelerated eyeblink conditioning are based on experiments using the same CS in fear conditioning preceding eyeblink conditioning in rodents (Neufeld and Mintz, 2001). The present findings extend the effects of preceding fear conditioning to CSs from two different modalities in humans. Accompanying autonomic responses decreased during eyeblink conditioning, which is also in line with the previous literature (Neufeld and Mintz, 2001).

In addition, prior fear conditioning resulted in changes of the topography of the conditioned responses. Responses were longer lasting, and frequently occurred at a shorter latency (Fig. 6), although the latter was not significant. Long-lasting, short-latency responses are well known in mouse eyeblink conditioning, and are thought to be at least partially driven by the amygdala (and not the cerebellum; Boele et al., 2010). Conditioned responses, on the other hand, showed some resemblance to responses that were discussed as being volitional in the early human eyeblink conditioning literature (for review, see Coleman and Webster, 1988). This does not exclude involvement of the amygdala and will be of interest to study in more detail in the future.

Our findings show that the modulatory effect of preceding fear conditioning, and therefore likely the amygdala, goes beyond the acquisition phase. Prior fear-conditioning and concomitant fear-extinction trials also impeded extinction of conditioned eyeblinks, which was accompanied by increased autonomic fear responses. Findings are in good agreement with the work by Farley et al. (2016), who showed that the modulatory effect of the amygdala is not restricted to acquisition, as predicted by the two-stage theory of aversive conditioning, but is also present during retention of conditioned eyeblinks. Emotional preconditioning has been found to enhance the eyeblink responses to the CS and to the US (Neufeld and Mintz, 2001). Concomitant presentation of fear and eyeblink CSs during extinction training has likely the same effect. The most parsimonious explanation is increased salience, that is aversiveness, of the CS (and US) input to the cerebellum, likely gated by the amygdala (Weisz et al., 1992; Taub and Mintz, 2010; Farley et al., 2016, 2018). The hypothesis that the amygdala gates selective attention to the CS rather than emotional modulation of responding is further supported by our observation that ratings of valence and arousal toward the fear CS, and of unpleasantness of the fear US were not different between groups. Likewise, eyeblink conditioning is facilitated by a stressful event before conditioning, which also leads to increased activity of the amygdala (Servatius et al., 2001; Shors, 2004; Weiss et al., 2005; Duncko et al., 2007). In fact, it has been shown by Steinmetz et al. (2017) that the facilitatory effect of the amygdala in eyeblink conditioning does not require memory formation in the amygdala, which, as outlined in the Introduction, is at variance with the two-stage theory of aversive conditioning.

Findings of impeded extinction of conditioned eyeblinks were observed regardless of the temporal presentation protocols (i.e., overlapping, alternating, or successive presentation of fear and eyeblink CS extinction trials); that is, effects were not restricted to the time of the fear CS presentation. Extinction training on day 2 lasted for a maximum of 26 min. Proposed effects of selective attention to the CS may be less only after more extended periods of extinction training (Itthipuripat et al., 2017).

One possible limitation of the present study is that eyeblink conditioning was acquired in a single session, and learning did not reach full saturation. Humans, however, acquire conditioned eyeblinks much faster than rodents (Spence, 1966). As stated above, the first conditioned eyeblink responses occur frequently within the first 10 paired CS/US trials (Kjell et al., 2018). Much of the learning is achieved at the end of a single session, and the additional increase in the incidence of CR across multiple sessions is comparatively small (Gerwig et al., 2010). Another possible limitation is that the interaction between conditioned fear and eyeblink responses was tested during extinction training, similar to studies testing additive response summation (Wolf, 1963; Weiss, 1964; Miller, 1969). Extinction of conditioned eyeblinks may have attenuated output of the cerebellar nuclei below threshold already after a limited number of trials. Magal and Mintz (2014) had mimicked eyeblink CRs by continuous stimulation of the cerebellar nuclei. The proposed third stage of learning, however, has never been tested directly during eyeblink conditioning in rodents.

Findings appear to contradict the observation that goal-directed eye movements may be beneficial in the treatment of posttraumatic stress disorder (see the “eye movement desensitization and reprocessing” (EMDR) method introduced by Shapiro, 1989). Eye movements involve the cerebellum, and it has been proposed that the cerebellum contributes to EDMR effects (Bergmann, 2000; Calancie et al., 2018). A recent fMRI study showed that EMDR in fact enhances the extinction of conditioned fear responses. This effect, however, was not specific to eye movements, but also occurred with an accompanying working memory task (de Voogd et al., 2018). The authors found that the activity of the amygdala was decreased, which was accompanied by altered connectivity with dorsolateral and ventromedial prefrontal pathways. Because of the known connections of the cerebellum with the dorsolateral and (shown more recently) ventromedial prefrontal areas, the cerebellum may well have a modulatory role in EDMR (Strick et al., 2009; Watson et al., 2009). The present data, however, show that a more direct inhibitory effect of the cerebellum on the amygdala may not be involved. Future studies, however, are needed to investigate other forms of cerebellar-dependent motor learning.

Experiments have been performed in men only. Results may be different in women particularly regarding learned fear responses (Lebron-Milad et al., 2012; Fenton et al., 2016). In rats, however, the role of the amygdala in eyeblink conditioning does not seem to be sex dependent (Bral et al., 2019). Furthermore, sex differences observed in eyeblink conditioning in rodents (Dalla and Shors, 2009) have not been replicated in humans (Wolf et al., 2009).

In conclusion, no evidence was found that cerebellum-dependent conditioned eyeblink responses accelerate extinction of conditioned fear. Rather, recall of conditioned fear was facilitated. As expected, fear conditioning facilitated subsequent eyeblink conditioning, but also impeded its extinction, accompanied by increased fear responses. Findings agree with the sensory gating hypothesis of the amygdala but are difficult to explain with the two-stage (or three-stage) theory of aversive conditioning, which would predict the suppression of conditioned fear once conditioned eyeblinks are acquired.

## Synthesis

Reviewing Editor: Christian Hansel, University of Chicago Decisions are customarily a result of the Reviewing Editor and the peer reviewers coming together and discussing their recommendations until a consensus is reached. When revisions are invited, a fact-based synthesis statement explaining their decision and outlining what is needed to prepare a revision will be listed below. The following reviewer(s) agreed to reveal their identity: John Freeman.

This interesting paper tests the idea that cerebellar conditioned responses can suppress conditioned fear responses. Authors tested 100 males in five different groups, each group having a different combination of fear-conditioning and eyeblink conditioning. They find that eyeblink CRs cannot facilitate extinction of fear CRs. However, fear CRs do impede extinction of eyeblink CRs, which is an important finding.

The manuscript presents the findings of an experiment designed to assess two competing theories of how the amygdala modulates cerebellar learning. Two-factor theories posit that fear conditioning develops in the amygdala early in training and then facilitates subsequent motor learning in the cerebellum by strengthening stimulus inputs or by modulating motivation. The sensory gating hypothesis is similar regarding initial training by positing that the amygdala increases stimulus inputs to the cerebellum. The two theories differ regarding the role of the amygdala after motor learning: two-factor theory proposes a third phase in which the cerebellum inhibits the amygdala and thereby causes extinction of fear conditioning; whereas the sensory gating theory proposes that the amygdala continues to modulate attention and the expression of motor learning after motor learning is well consolidated. The authors examined this difference in the theories by giving concurrent fear conditioning and eyeblink conditioning during extinction training in humans. Participants were trained in differential fear conditioning, followed by eyeblink conditioning. They were then separated into three groups that received different arrangements of fear and eyeblink extinction trials and were compared to groups that only received fear or eyeblink conditioning. The general results are that groups with mixed fear and eyeblink conditioning trials showed more fear and eyeblink CRs relative to the control groups. The results are not consistent with the particular two-factor theory being tested since cerebellar output during eyeblink CRs should suppress the amygdala and therefore decrease fear CRs. In contrast, eyeblink and fear CRs were higher than in controls, suggesting that the amygdala was not being inhibited by the cerebellum. The methods and experimental design are very good. The authors acknowledge some limitations of their experiment in the discussion and interpret the results appropriately.

Major points:

Introduction:

- The authors state that “As suggested by Magal and Mintz (2014), this suppression may help to facilitate fear extinction which is at the core of exposure therapy in the treatment of many anxiety disorders (Craske and Mystkowski, 2006; Craske et al., 2014 for reviews).”

This Reviewer does not see immediately how this is clinically relevant or advantageous. The amygdala would only be inactivated during the CS period, which is typically only a few hundred of milliseconds. To our knowledge, no study ever reported long lasting amygdala inhibition as a result of eyeblink conditioning. Moreover, it is not a given that the amygdala is also inactivated by an eyeblink CR when the eyeblink CS is paired with the aversive stimulus (fear US) that triggers the fear response. Please address.

- Indeed, the Freeman group suggests sensory gating by the amygdala.

Can the authors speculate more about the mechanisms and anatomical pathways involved in amygdala sensory gating of cerebellar learning?

Methods:

- Because menstrual cycle and oral contraceptives influence acquisition and extinction of conditioned fear (Merz et al., 2018), only male participants were included in this study.

Authors only include males, but the rest of the paper reads as if their findings apply to both genders. Either mention explicitly in discussion that this study only involved male participants and discuss this more thoroughly, or maybe even add to title: “...in males”.

Results:

- Overall, the eyeblink and fear-conditioning data is not transparent. Authors only show averages with SE. Only showing averaged values with a standard error is absolutely outdated. We encourage the authors to show individual datapoints (see current ongoing discussions in field of neuroscience about data transparency).

Fig 4: The green color scheme is too subtle. Unilateral SE is not accepted. Panel D: bar plots {plus minus} SE are obsolete and should be replaced with box plots and showing individual datapoints.

Authors should show trial-by-trial analysis of first block. In the block average the fear-trained groups already have a CR incidence of about 30%, which is very high.

Authors only show CR incidence, please provide more details about amplitude and timing of the eyeblink CRs.

Why do the authors not look at the timing of CRs? It is known from mouse studies that there is an amygdala-driven eyelid response, which typically has a much shorter latency to CR peak than the cerebellar CR (see Boele et al., 2010). It is not unlikely that the high CR incidence in this study is caused by these a similar type of response. Showing the actual eyeblink traces would help the reader enormously with interpreting this dataset.

The authors should indicate why partial reinforcement was used for the fear and eyeblink conditioning procedures. I believe this was used to slow the rate of extinction a bit.

During extinction, there were three groups with different fear vs. eyeblink trial arrangements: overlapping, alternating, and subsequent. The reasons for using these different procedures was not indicated. The authors should why each group was used and how they relate to testing the theories, starting on page 7.

The “subsequent” group might be called “sequential” or “successive” to indicate that fear trials were given followed by eyeblink trials. This label might make the procedure for this group more intuitive.

The figures indicate some of the significant differences, but not all of them. This causes a little bit of confusion and requires the reader to repeatedly go back and forth between the text and figures to assess the significant effects. I suggest that the authors develop ways to indicate significant comparisons in all of the panels of the figures, since there are many important differences that should be highlighted (e.g., extinction data in Figure 5).

In the results section, the data analyses jump from Figure 4 to Figure 5 and back to Figure 4, which makes it a little difficult to follow. The authors might consider presenting all of the SCR data during eyeblink conditioning (Figure 5) after the acquisition and extinction data for eyeblink conditioning (Figure 4).

Questionnaires were given to participants regarding CS valence, arousal, and the unpleasantness of the US. No rationale was provided for why these particular ratings were collected. Presumably, these measures were included to assess the three-factor theory.

The finding of no group differences in arousal or unpleasantness of the US is a potentially important finding because it suggests that the group differences in extinction are not related to these measures. My reading of the paper suggests that these data provide additional evidence in support of a CS gating (attentional) hypothesis rather than emotional modulation of responding, but the authors might not agree. Either way, some additional discussion of these results is warranted.

If differences between the three experimental groups were predicted/hypothesized (see comment 2), a discussion of why they did not differ much in extinction should be added to the discussion section.

## Author Response

Significance Statement Comments for Author (Required):

I did not see one yet.

Authors’ response:

The significance statement has been uploaded.

Comments on the Visual Abstract for Author (Required):

N/A

Synthesis Statement for Author (Required):

This interesting paper tests the idea that cerebellar conditioned responses can suppress conditioned fear responses. Authors tested 100 males in five different groups, each group having a different combination of fear-conditioning and eyeblink conditioning. They find that eyeblink CRs cannot facilitate extinction of fear CRs. However, fear CRs do impede extinction of eyeblink CRs, which is an important finding.

The manuscript presents the findings of an experiment designed to assess two competing theories of how the amygdala modulates cerebellar learning. Two-factor theories posit that fear conditioning develops in the amygdala early in training and then facilitates subsequent motor learning in the cerebellum by strengthening stimulus inputs or by modulating motivation. The sensory gating hypothesis is similar regarding initial training by positing that the amygdala increases stimulus inputs to the cerebellum. The two theories differ regarding the role of the amygdala after motor learning: two-factor theory proposes a third phase in which the cerebellum inhibits the amygdala and thereby causes extinction of fear conditioning; whereas the sensory gating theory proposes that the amygdala continues to modulate attention and the expression of motor learning after motor learning is well consolidated. The authors examined this difference in the theories by giving concurrent fear conditioning and eyeblink conditioning during extinction training in humans. Participants were trained in differential fear conditioning, followed by eyeblink conditioning. They were then separated into three groups that received different arrangements of fear and eyeblink extinction trials and were compared to groups that only received fear or eyeblink conditioning. The general results are that groups with mixed fear and eyeblink conditioning trials showed more fear and eyeblink CRs relative to the control groups. The results are not consistent with the particular two-factor theory being tested since cerebellar output during eyeblink CRs should suppress the amygdala and therefore decrease fear CRs. In contrast, eyeblink and fear CRs were higher than in controls, suggesting that the amygdala was not being inhibited by the cerebellum. The methods and experimental design are very good. The authors acknowledge some limitations of their experiment in the discussion and interpret the results appropriately.

Authors’ response:

We wish to thank the reviewers for their favorable assessment. Based on their insightful and very helpful comments we have carefully revised our manuscript as outlined in detail below.

New: eNeuro is launching a new effort to improve rigor, transparency, and reproducibility, with a strong encouragement to use estimation statistics. Please refer to the editorial

Protected link and commentary Protected link for the why and how. If the use of estimation statistics applies to your research, you may consider including it for the revision. We will regularly highlight these papers to the community to show how helpful estimation statistics can be, increasing the exposure of your work.

Authors’ response:

We followed the recommendation of eNeuro and have included estimation statistics in the revision of our manuscript (see Figures 4, 6, 4-1 and 5-1).

Major points:

Introduction:

- The authors state that “As suggested by Magal and Mintz (2014), this suppression may help to facilitate fear extinction which is at the core of exposure therapy in the treatment of many anxiety disorders (Craske and Mystkowski, 2006; Craske et al., 2014 for reviews).”

This Reviewer does not see immediately how this is clinically relevant or advantageous. The amygdala would only be inactivated during the CS period, which is typically only a few hundred of milliseconds. To our knowledge, no study ever reported long lasting amygdala inhibition as a result of eyeblink conditioning. Moreover, it is not a given that the amygdala is also inactivated by an eyeblink CR when the eyeblink CS is paired with the aversive stimulus (fear US) that triggers the fear response. Please address.

Authors’ response:

These points are well taken. The introduction has been revised accordingly

- Indeed, the Freeman group suggests sensory gating by the amygdala.

Can the authors speculate more about the mechanisms and anatomical pathways involved in amygdala sensory gating of cerebellar learning?

Authors’ response:

Done. Current knowledge about the involved anatomical pathways and some more details about the proposed mechanisms have been added to the introduction.

Methods:

- Because menstrual cycle and oral contraceptives influence acquisition and extinction of conditioned fear (Merz et al., 2018), only male participants were included in this study.

Authors only include males, but the rest of the paper reads as if their findings apply to both genders. Either mention explicitly in discussion that this study only involved male participants and discuss this more thoroughly, or maybe even add to title: “...in males”.

Authors’ response:

Title, abstract and discussion have been changed accordingly. It is now explicitly mentioned and discussed in more detail that the study has been performed in men.

Results:

- Overall, the eyeblink and fear-conditioning data is not transparent. Authors only show averages with SE. Only showing averaged values with a standard error is absolutely outdated. We encourage the authors to show individual datapoints (see current ongoing discussions in field of neuroscience about data transparency).

Fig 4: The green color scheme is too subtle. Unilateral SE is not accepted. Panel D: bar plots {plus minus} SE are obsolete and should be replaced with box plots and showing individual datapoints.

Authors’ response:

These points are well taken. Figure 4 has been revised accordingly (see Fig. 5 in the revised manuscript). We decided to use the same, single green color code for Groups 1, 2 and 3 because each group is compared to the control Group 5 (shown in yellow) in individual plots. Instead of unilateral SE the 95% confidence intervals are shown. In addition, individual data points are shown. Panel D has been replaced by a Cumming estimation plot (Fig. 6 in the revised manuscript). All other figures in the revised manuscript have been changed accordingly.

Authors should show trial-by-trial analysis of first block. In the block average the fear-trained groups already have a CR incidence of about 30%, which is very high.

Authors’ response:

Done. Trial-by-trial analysis of the first acquisition block has been included in the Extended Data (Figure 5-2) of the revised manuscript. Trial-by-trial analysis further illustrates that CR incidences were higher in the three groups which had received prior fear conditioning (Groups 1-3, indicated in green) compared to the control group (Group 5, indicated in yellow). Of note, and outlined in the discussion, human participants often present with conditioned responses already in the first block of paired conditioned responses (e.g. Kjell et al., 2018; see also Fig. 2 in Smit et al., 2008).

Kjell K, Lowgren K, Rasmussen A (2018) A Longer Interstimulus Interval Yields Better Learning in Adults and Young Adolescents. Frontiers in Behavioral Neuroscience 12:299.

Smit AE, van der Geest JN, Vellema M, Koekkoek SK, Willemsen R, Govaerts LC, Oostra BA, De Zeeuw CI, VanderWerf F (2008) Savings and extinction of conditioned eyeblink responses in fragile X syndrome. Genes, Brain and Behavior 7:770-777.

Authors only show CR incidence, please provide more details about amplitude and timing of the eyeblink CRs.

Why do the authors not look at the timing of CRs? It is known from mouse studies that there is an amygdala-driven eyelid response, which typically has a much shorter latency to CR peak than the cerebellar CR (see Boele et al., 2010). It is not unlikely that the high CR incidence in this study is caused by these a similar type of response. Showing the actual eyeblink traces would help the reader enormously with interpreting this dataset.

Authors’ response:

The authors like to thank the reviewer for his/her insightful comment. We carefully reexamined our raw data. The reviewer is correct, participants in the three groups that had received fear conditioning prior to eyeblink conditioning tended to show more shorter latency responses than participants in the control group. This is illustrated in an additional Figure showing EMG eyeblink traces of individual subjects from each group (Fig. 6). Timing of conditioned responses, however, showed a high degree of variability in each of the four groups, and neither CR onset nor CR peaktime showed a significant difference between groups. The only significant change we observed was regarding CR onset in unpaired CS only trials which showed a significant change across acquisition blocks (Fig. 5.3 in Extended Data). CR onset had an earlier onset in later acquisition trials, a finding that is in accordance with the literature (e.g. Gruart et al., 2000; Koekkoek et al., 2002). This shift towards the onset of the CS (not the US) was present in all groups but was not found in paired CS/US acquisition trials. CR peak time, however, did not show a significant block effect in the acquisition phase neither in paired nor in unpaired trials.

Examination of the raw data, however, suggested that participants show different types of responses in the CS-US window: alpha responses, but also shorter latency responses which often lasted until the onset of the US, and better timed (closed to US onset) responses. The shorter latency responses showed some resemblance to responses which were discussed as being volitional in the early human eyeblink conditioning literature (Coleman and Webster, 1988, for review). Because the shorter latency responses appeared to occur more frequently in the experimental groups (groups 1, 2 and 3), we agree with the reviewer, that they may be driven by the amygdala - and may therefore bear similarities with the SLR described in mice (Boele et al., 2010). It will be of interest to study in more detail in the future to what extent these shorter latency responses are volitional and/or driven by the amygdala.

In fact, although analysis of CR onset and peak time latencies showed no differences between groups, CR duration and area were significantly increased in unpaired CS only trials in the experimental groups compared to the control group, indicating that responses with a long response duration were more frequent in the first. Data on CR onset, peaktime, duration and area are shown in Extended Data of the revised manuscript. Results and Discussion have been revised accordingly.

Note that reevaluation of individual data revealed a minor mistake in the assignment of CR responses to the different learning blocks. Statistics on CR incidences have therefore been recalculated. Results did not change. The accurate numbers are presented in the revised manuscript (and have been highlighted accordingly).

We like to thank Henk-Jan Boele and Anders Rasmussen for helpful discussion on shorter latency responses in humans.

Boele HJ, Koekkoek SK, De Zeeuw CI (2010) Cerebellar and extracerebellar involvement in mouse eyeblink conditioning: the ACDC model. Frontiers in Cellular Neuroscience 3:19.

Gruart A, Schreurs BG, del Toro ED, Delgado-García JM (2000) Kinetic and frequency-domain properties of reflex and conditioned eyelid responses in the rabbit. Journal of Neurophysiology 83:836-852.

Koekkoek SK, Den Ouden WL, Perry G, Highstein SM, De Zeeuw CI (2002) Monitoring kinetic and frequency-domain properties of eyelid responses in mice with magnetic distance measurement technique. Journal of Neurophysiology 88:2124-2133.

Coleman SR, Webster S (1988) The problem of volition and the conditioned reflex. Part II. Voluntary-responding subjects, 1951-1980. Behaviorism 16:17-49.

The authors should indicate why partial reinforcement was used for the fear and eyeblink conditioning procedures. I believe this was used to slow the rate of extinction a bit.

Authors’ response:

Correct. Methods have been revised accordingly.

During extinction, there were three groups with different fear vs. eyeblink trial arrangements: overlapping, alternating, and subsequent. The reasons for using these different procedures was not indicated. The authors should why each group was used and how they relate to testing the theories, starting on page 7.

Authors’ response:

Magal and Mintz (2014) had shown that output of the cerebellar nuclei to the amygdala was suppressed during presentation of an aversive US. As indicated above, one may expect that effects are most prominent during the time of the presentation of the aversive stimulus. Thus, in case the two (or three) stage theory of learning was correct, and effects were not lasting, the proposed modulatory effects on the amygdala may be expected to be most prominent during the presentation of the eyeblink CS. Hence, effects on extinction of learned fear responses may be expected to be more prominent in Group 1 (“overlapping extinction trials”) compared to Group 2 (“alternating extinction trials”). Groups 3, 4 and 5 were control groups. This information has been added to the revised methods.

Magal A, Mintz M (2014) Inhibition of the amygdala central nucleus by stimulation of cerebellar output in rats: a putative mechanism for extinction of the conditioned fear response. European Journal of Neuroscience 40:3548-3555.

The “subsequent” group might be called “sequential” or “successive” to indicate that fear trials were given followed by eyeblink trials. This label might make the procedure for this group more intuitive.

Authors’ response:

Done. The “subsequent” group is called “successive” in the revised manuscript as recommended.

The figures indicate some of the significant differences, but not all of them. This causes a little bit of confusion and requires the reader to repeatedly go back and forth between the text and figures to assess the significant effects. I suggest that the authors develop ways to indicate significant comparisons in all of the panels of the figures, since there are many important differences that should be highlighted (e.g., extinction data in Figure 5).

Authors’ response:

Done. Significant differences are indicated in Figures 4, 6, 7, 4-1, 5-2 and 5-6 in the revised version of the manuscript as suggested.

In the results section, the data analyses jump from Figure 4 to Figure 5 and back to Figure 4, which makes it a little difficult to follow. The authors might consider presenting all of the SCR data during eyeblink conditioning (Figure 5) after the acquisition and extinction data for eyeblink conditioning (Figure 4).

Authors’ response:

We thank the reviewer for this helpful suggestion. In the revised manuscript, we have changed the order of results as suggested.

Questionnaires were given to participants regarding CS valence, arousal, and the unpleasantness of the US. No rationale was provided for why these particular ratings were collected. Presumably, these measures were included to assess the three-factor theory.

Authors’ response:

Verbal responses of subjective experiences were used as additional outcome parameters. In the human fear conditioning research literature, verbal ratings about CS valence and arousal are well established to assess the affective components of learning (Lonsdorf et al., 2017, for review). Because US salience (same as CS salience) has a direct impact on acquisition of learned fear, unpleasantness of the US is commonly rated after fear acquisition training to control for possible effects of habituation and sensitization (Lonsdorf et al., 2017, for review). This information has been added to the revised methods.

Lonsdorf TB et al. (2017) Don’t fear ‘fear conditioning’: Methodological considerations for the design and analysis of studies on human fear acquisition, extinction, and return of fear. Neuroscience and Biobehavioral Reviews 77:247-285.

The finding of no group differences in arousal or unpleasantness of the US is a potentially important finding because it suggests that the group differences in extinction are not related to these measures. My reading of the paper suggests that these data provide additional evidence in support of a CS gating (attentional) hypothesis rather than emotional modulation of responding, but the authors might not agree. Either way, some additional discussion of these results is warranted.

Authors’ response:

This is a very good point. We like to thank the reviewer for his/her helpful suggestion. We agree with the interpretation of the reviewer and added this aspect to the revised discussion.

If differences between the three experimental groups were predicted/hypothesized (see comment 2), a discussion of why they did not differ much in extinction should be added to the discussion section.

Authors’ response:

The reviewer is correct, findings of impeded extinction of conditioned eyeblinks were observed regardless of the temporal presentation protocols (that is, overlapping, alternating or successive presentation of fear and eyeblink CS extinction trials). Thus, effects of concomitant fear conditioning were longer lasting, and not restricted to the time of the fear CS presentation. Extinction training on day 2 lasted for a maximum of 26 minutes. The proposed effects of selective attention to the CS may be less only after more extended periods of extinction training (e.g. Itthipuripat et al. (2017)). We revised the discussion accordingly.

Itthipuripat S, Cha K, Byers A, Serences JT (2017) Two different mechanisms support selective attention at different phases of training. PLOS Biology 15:e2001724.
