# Supplementary figures and images for: Interaction of Fear Conditioning with Eyeblink Conditioning Supports the Sensory Gating Hypothesis of the Amygdala in Men
Source: eNeuro. 2020 Sep 23;7(5):ENEURO.0128-20.2020. doi: 10.1523/ENEURO.0128-20.2020 (PMC7559307; doi:10.1523/ENEURO.0128-20.2020)

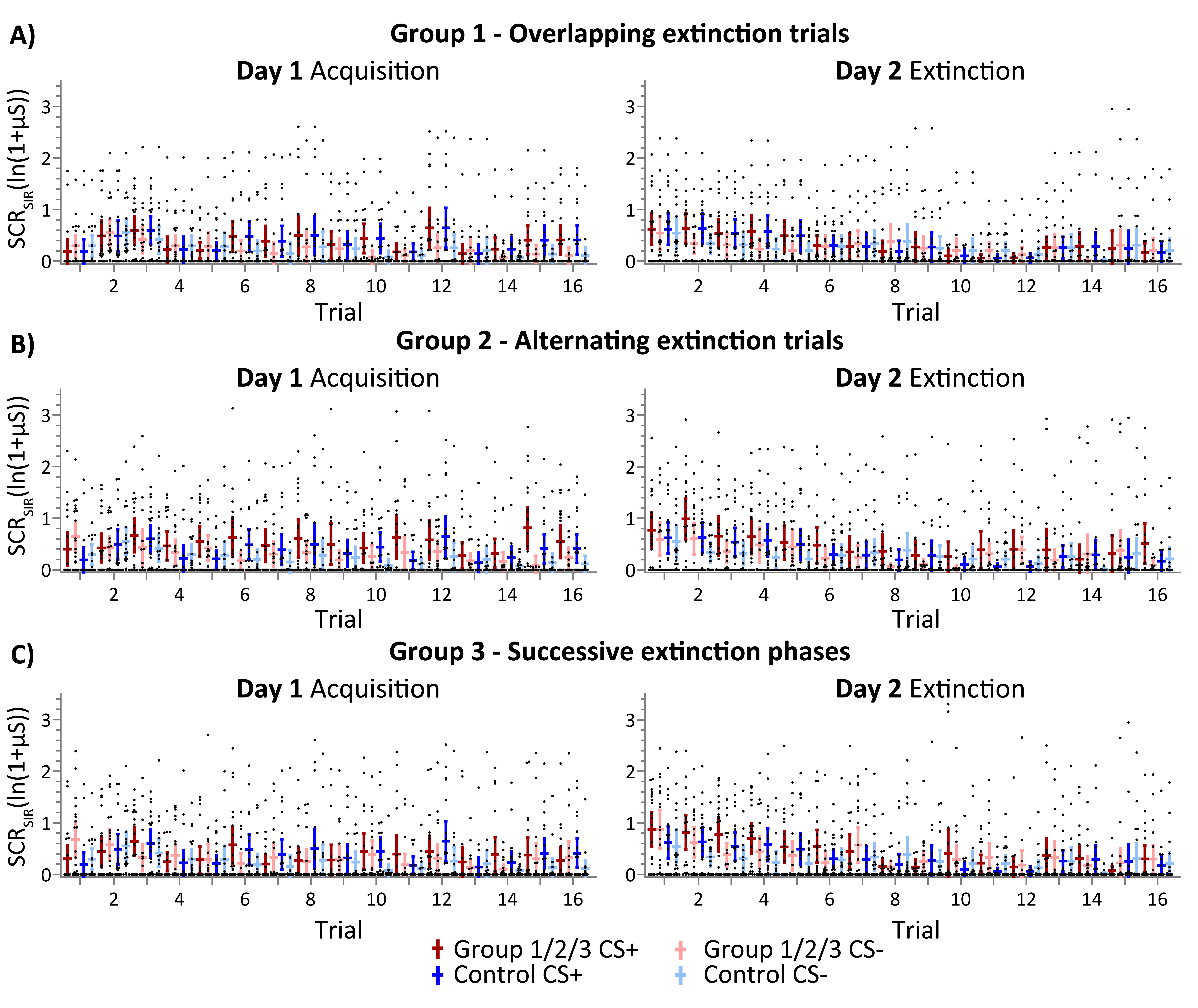

Supplement: Figure 3-1 — Fear conditioning data considering SCRSIR. Group mean SCRSIR and individual data on day 1 (fear acquisition training) and day 2 (extinction training) across trials. (A) Group 1 (“overlapping extinction trials”; shown in red) vs. Group 4 (control; shown in blue), (B) Group 2 (“alternating extinction trials”; shown in red) vs. Group 4 (control; shown in blue), (C) Group 3 (“successive extinction phases”, shown in red) vs. Group 4 (control; shown in blue). Horizontal lines represent mean values, vertical lines indicate 95% confidence intervals. Black dots show individual data points. Fear acquisition training: SCRSIR was significantly higher in CS+ trials compared to CS- trials in all groups (A-C). Non-parametric ANOVA-type statistics revealed a significant main effect of Trial (trial 1-16; F12 = 8.29, p < 0.001), Stimulus type (CS+ vs. CS-; F1 = 9.53, p = 0.0028) and a significant Trial x Stimulus type interaction (F12.1 = 6.24, p < 0.001). No significant main effect of Group (p = 0.8), and no significant Group x Stimulus (p = 0.24), Group x Trial (p = 0.82) or Group x Stimulus x Trial (p = 0.71) interactions were revealed. Fear extinction training: SCRSIR were higher in CS+ trials compared to CS- trials in all groups at the beginning of extinction training phase. This difference disappeared in later trials (A-C). Non-parametric ANOVA-type statistics revealed a significant main effect of Trial (F11.2 = 13.70, p < 0.001), Stimulus type (F1 = 5.58, p = 0.0207) and a significant Trial x Stimulus type interaction (F12 = 2.17, p = 0.0112). The Group main effect (p = 0.49), and the Group x Stimulus (p = 0.18), Group x Trial (p = 0.14) and Group x Stimulus x Trial (p = 0.79) interactions were not significant. Download Figure 3-1, TIF file. [file enu-eN-NWR-0128-20-s01.tif]

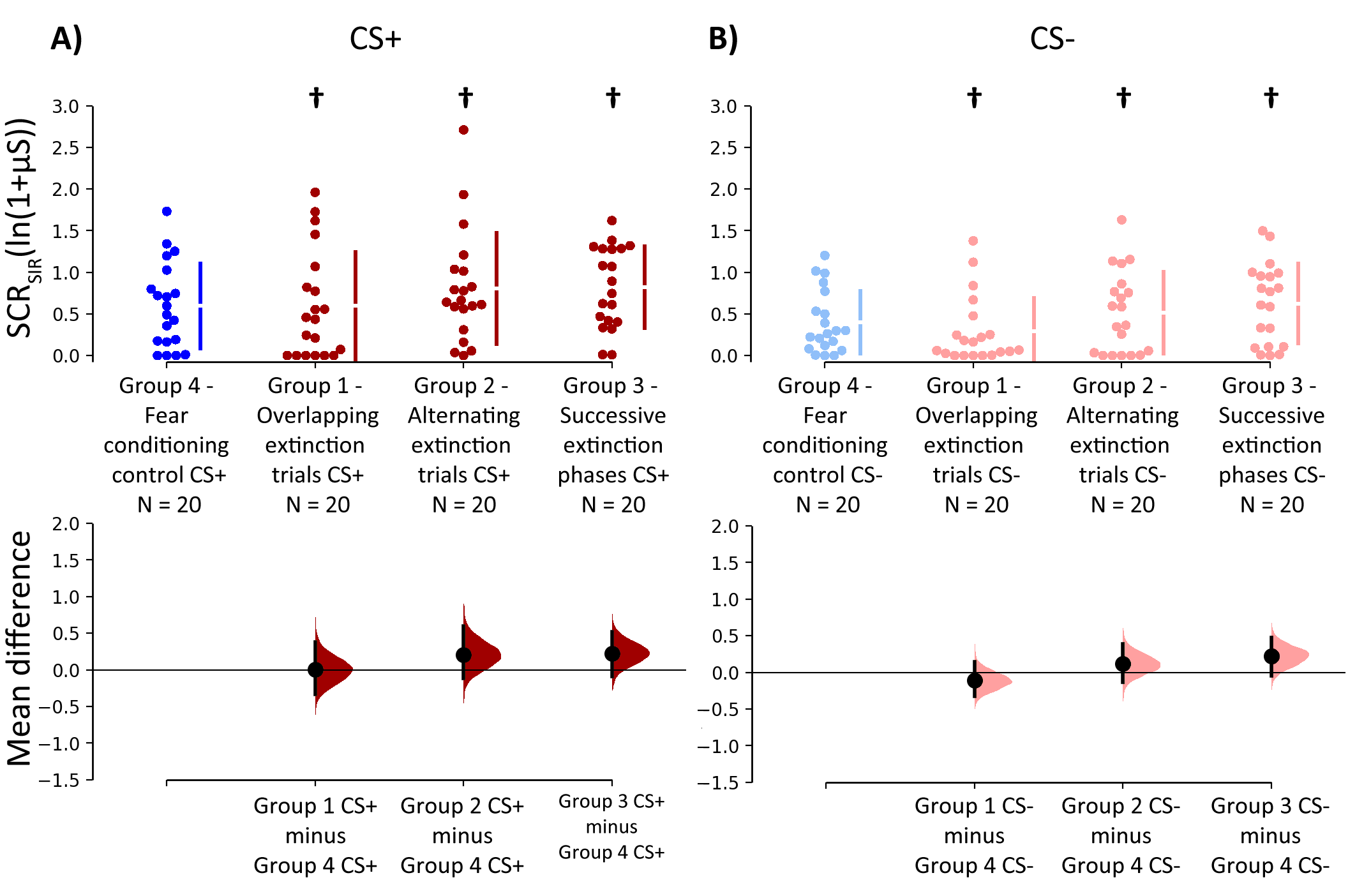

Supplement: Figure 4-1 — Recall of learned fear responses at the beginning of the extinction phase. Cumming estimation plots showing mean differences between groups 1-3 (shown in red) and the control group 4 (shown in blue) averaged across the first three extinction trials of A) CS+ and B) CS- SCRSIR. Dots on upper panel represent individual data points. Gapped lines indicate group means (gap) and standard deviations. Lower panel shows effects sizes. Black dots represent mean differences between groups and error bars 95% confidence intervals (CI). 95% CI are calculated by bootstrap resampling (Ho et al., 2019). Filled curves represent the bootstrap sampling distribution of the observed data. Dark colors = CS+, light colors = CS. † indicates significant SCRSIR differences between CS+ and CS- in the same group (Wilcoxon signed rank test, p < 0.05). Mann-Whitney U tests revealed no significant differences comparing Group 1 (CS+: U = 187, Z = -0.354, p = 0.72; CS-: U = 144, Z = -1.517, p = 0.13), Group 2 (CS+: U = 166, Z = -0.907, p = 0.37; CS-: U = 187, Z = -0.352, p = 0.73), and Group 3 (CS+: U = 143, Z = -1.542, p = 0.12; CS-: U = 148, Z = -1.407, p = 0.159) with Group 4, respectively. Download Figure 4-1, TIF file. [file enu-eN-NWR-0128-20-s02.tif]

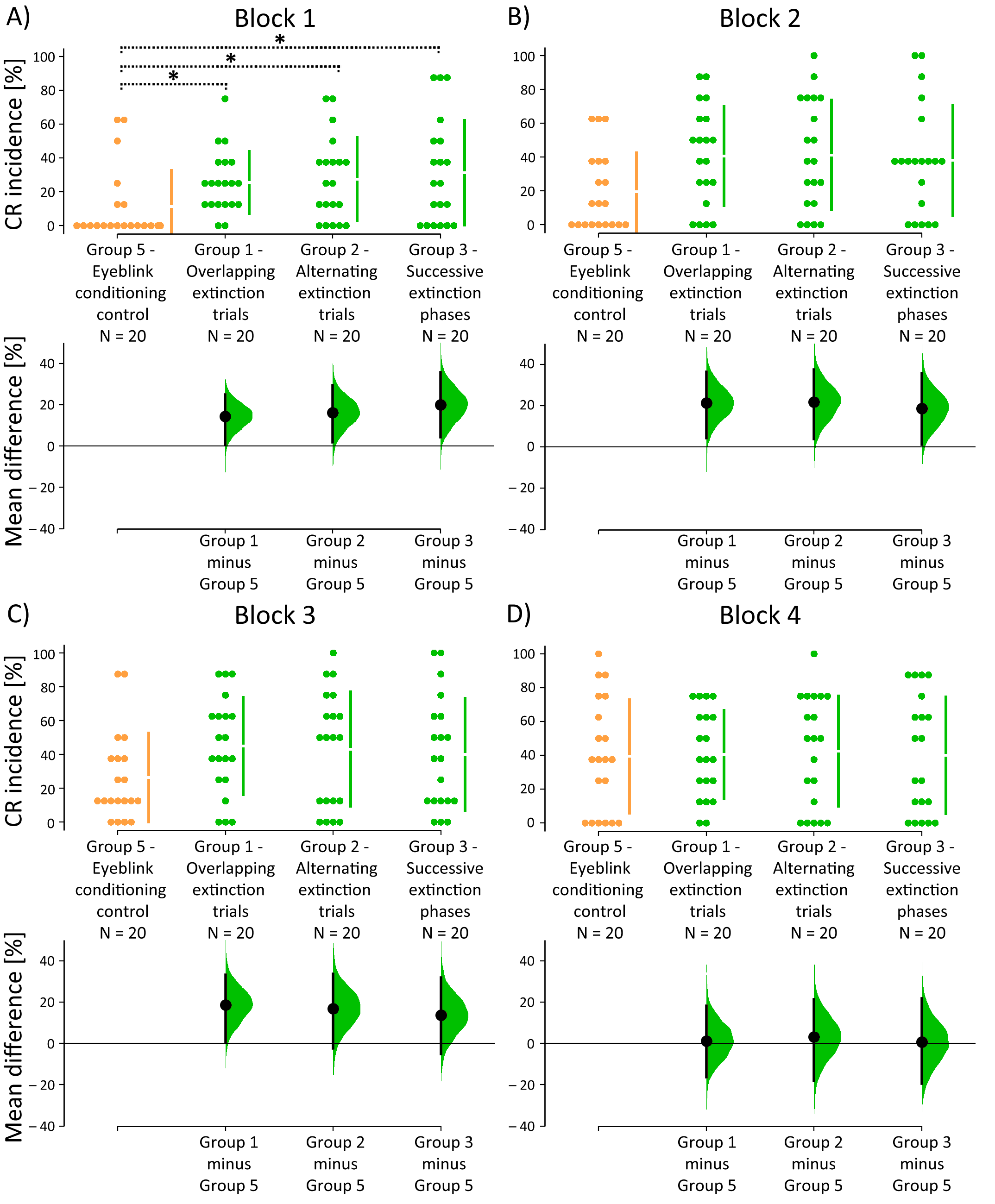

Supplement: Figure 5-1. — CR incidences shown for the first four acquisition blocks (block à eight trials). Cumming estimation plots showing mean differences between groups 1-3 (shown in green) and control group 5 (shown in yellow) for acquisition blocks A) 1, B) 2, C) 3 and D) 4. Dots on upper panel represent individual data points. Gapped lines indicate group means (gap) and standard deviations. Lower panel shows effects sizes. Black dots represent mean differences between groups and error bars 95% confidence intervals (CI). 95% CI are calculated by bootstrap resampling (Ho et al., 2019). Filled curves represent the bootstrap sampling distribution of the observed data. * indicates significant differences between respective stimuli between Groups 1/2/3 and Group 5 (least square means tests, p values < 0.05). Non-parametric ANOVA-type statistics performed in individual blocks revealed significant group differences in the first block only (Block 1: F3 = 3.87, p = 0.0124; Block 2: F3 = 2.51, p = 0.0646; Block 3: F3 = 1.49, p = 0.2551; Blocks 4-10 p > 0.41). Post-hoc pairwise comparisons revealed significantly increased CR incidences in Groups 1, 2 and 3 compared to the Group 5 (control) during the first acquisition block (least square means tests, all p values < 0.0086. Download Figure 5-1, TIF file. [file enu-eN-NWR-0128-20-s03.tif]

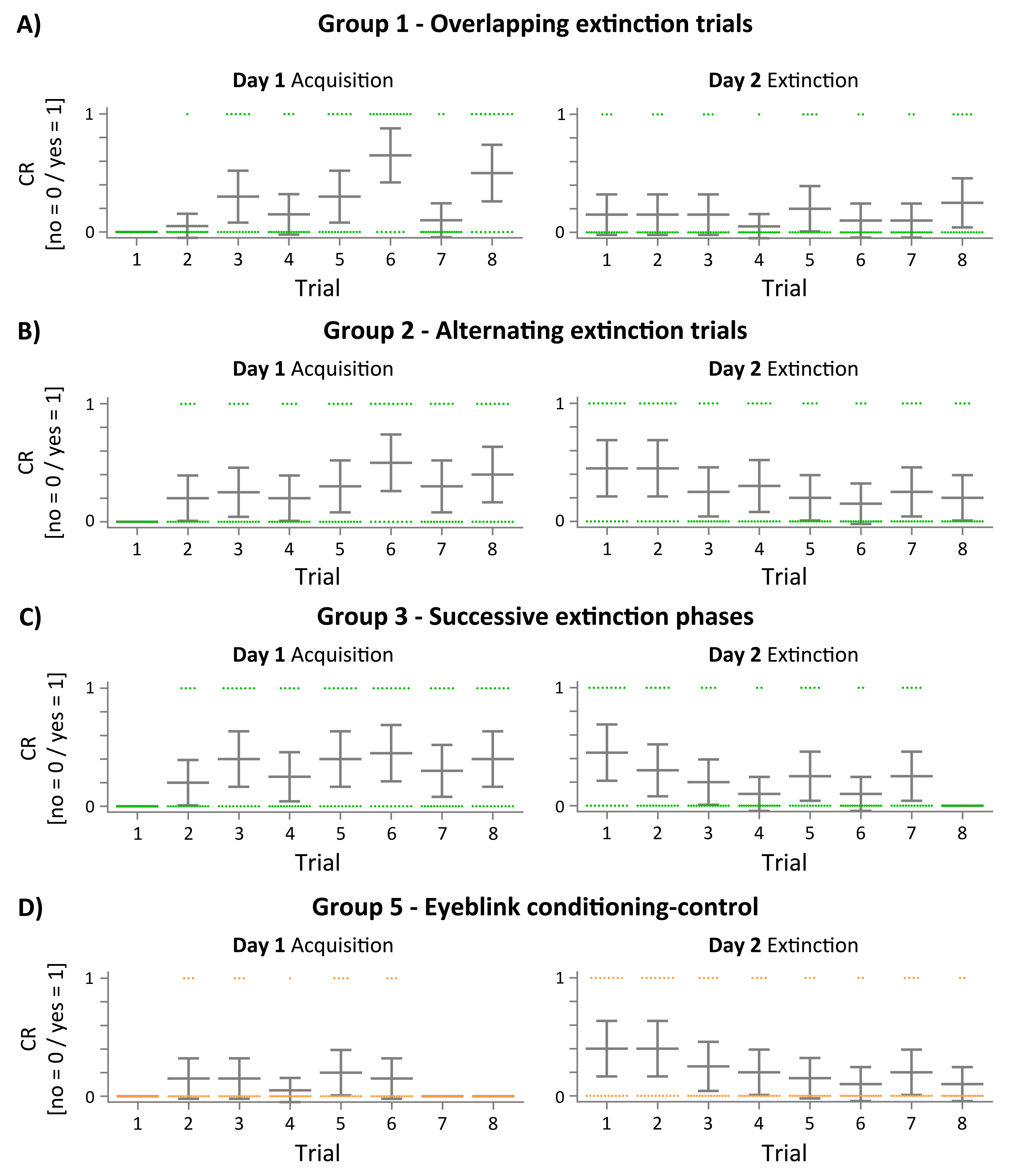

Supplement: Figure 5-2. — Trial-by-trial analysis of CR incidences considering the first acquisition block and the first extinction block (blocks à eight trials). The presence of a CR in an individual trial in each participant was coded as 1, and the absence was coded as 0. (A) Group 1 (“overlapping extinction trials”); (B) Group 2 (“alternating extinction trials”); (C) Group 3 (“successive extinction phases”); (D) Group 5 (“Eyeblink conditioning-control”). Horizontal lines represent mean probability in each group that a CR occurred in a given trial, vertical lines indicate 95% confidence intervals. Colored dots show individual data points. Download Figure 5-2, TIF file. [file enu-eN-NWR-0128-20-s04.tif]

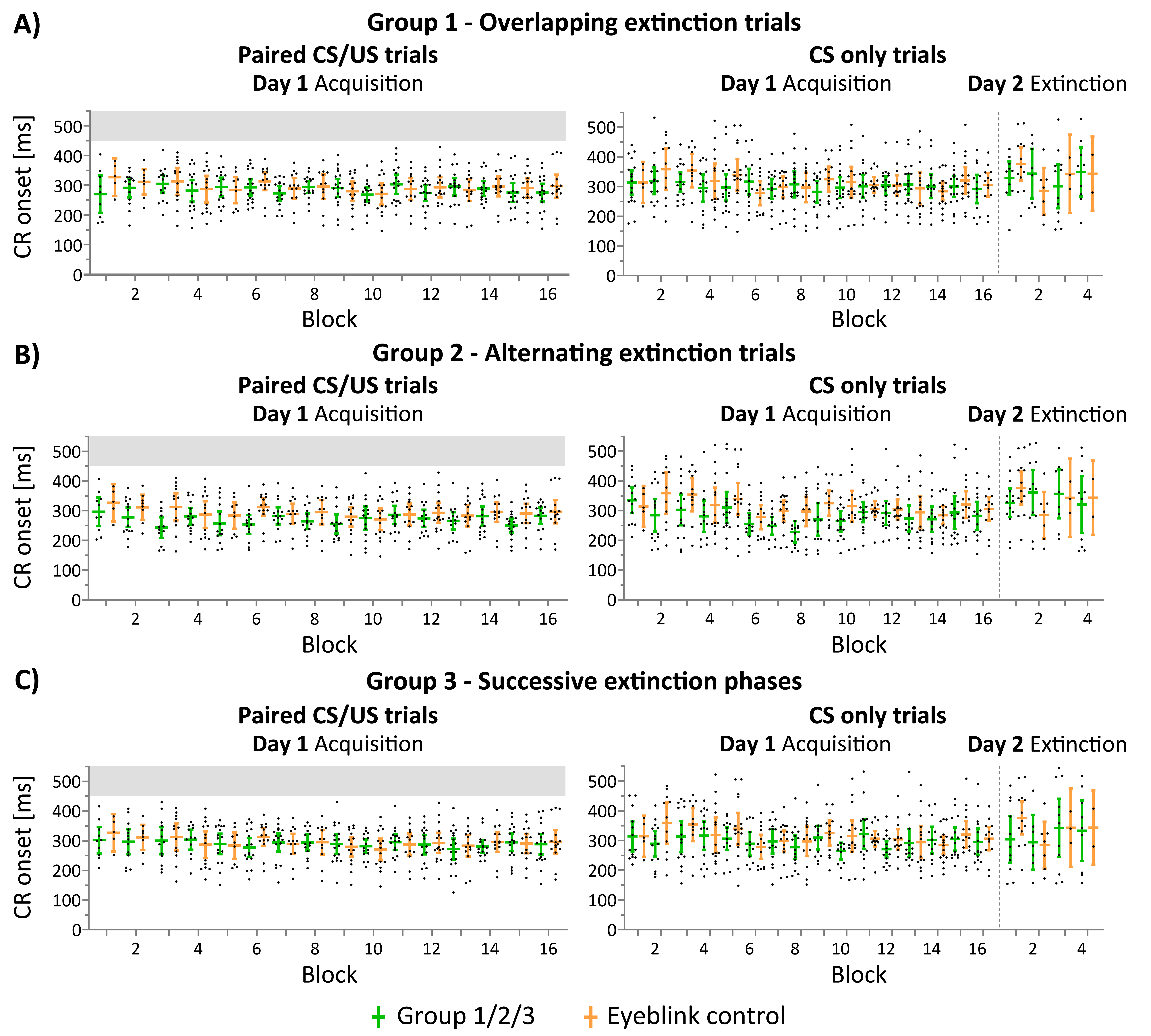

Supplement: Figure 5-3. — Eyeblink conditioning: CR onset. CR onset latencies are expressed as time after CS onset. Grey shading represents the time of the US presentation in CS/US paired trials. Group mean CR onset latencies and individual data are shown in the acquisition phase on day 1 (16 blocks à 8 trials corresponding to 5 CS/US trials and 3 CS only trials per block), and in the extinction phase on day 2 (4 blocks à 8 trials). (A) Group 1 (“overlapping extinction trials”, shown in green) vs. Group 5 (control, shown in yellow); (B) Group 2 (“alternating extinction trials”, shown in green) vs. Group 5 (control, shown in yellow); (C) Group 3 (“successive extinction phases”, shown in green) vs. Group 5 (control, shown in yellow). Horizontal lines represent mean values, vertical lines indicate 95% confidence intervals. Black dots show individual data points. Acquisition phase: In paired CS/US trials, non-parametric ANOVA-type statistics revealed no significant effects of Block (p = 0.12), Group (p = 0.48) or Group x Block interactions (p = 0.58). In CS only trials, non-parametric ANOVA-type statistics revealed a significant effect of Block (F11 = 1.83; p = 0.047) - reflecting an earlier onset in later acquisition trials, that is a shift towards CS onset, a finding that is in accordance with the literature (e.g. Gruart et al., 2000; Koekkoek et al., 2002), but no Group (p = 0.36) or Group x Block interaction effects (p = 0.58). Extinction phase: Non-parametric ANOVA-type statistics revealed no significant main effect of Block (p = 0.49), Group (p = 0.72) or Group x Block interactions (p = 0.69). Download Figure 5-3, TIF file. [file enu-eN-NWR-0128-20-s05.tif]

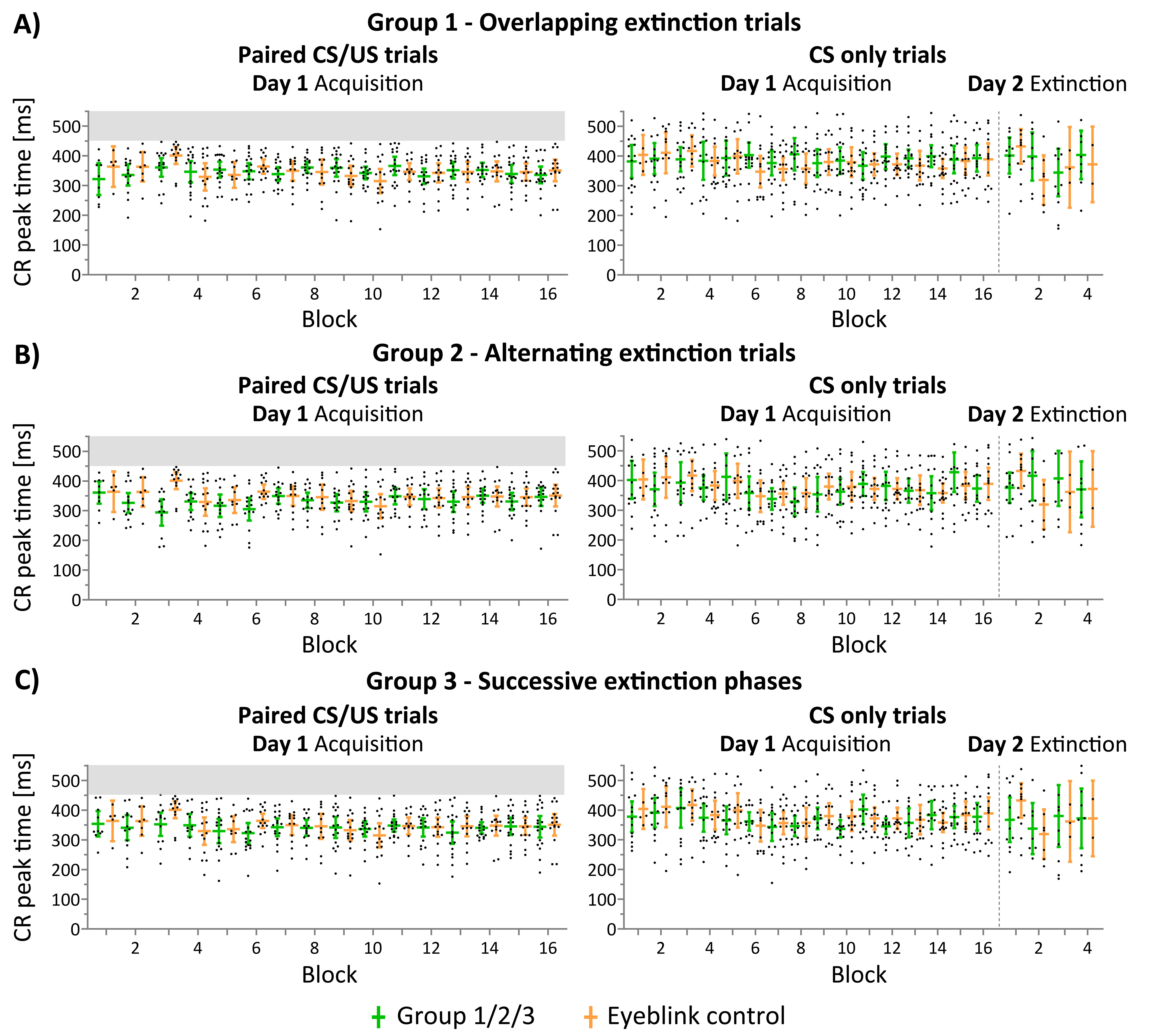

Supplement: Figure 5-4. — Eyeblink conditioning: CR peak time. Peak time latencies are expressed as time after CS onset. Grey shading represents the time of the US presentation in CS/US paired trials. In paired CS/US trials, CR peak time was defined at the time of maximum amplitude before US onset. In unpaired CS only trials, CR peak time was defined at the time of the maximum CR amplitude. Group mean CR peak time latencies and individual data are shown in the acquisition phase on day 1 (16 blocks à 8 trials corresponding to 5 CS/US trials and 3 CS only trials per block), and in the extinction phase on day 2 (4 blocks à 8 trials). (A) Group 1 (“overlapping extinction trials”, shown in green) vs. Group 5 (control, shown in yellow); (B) Group 2 (“alternating extinction trials”, shown in green) vs. Group 5 (control, shown in yellow); (C) Group 3 (“successive extinction phases”, shown in green) vs. Group 5 (control, shown in yellow). Horizontal lines represent mean values, vertical lines indicate 95% confidence intervals. Black dots show individual data points. Acquisition phase: Non-parametric ANOVA-type statistics revealed no significant main effect of Block (CS/US paired trials p = 0.27, CS only trials: p = 0.18), Group (CS/US paired trials p = 0.67, CS only trials: p = 0.57) or Group x Block interactions (CS/US paired trials p = 0.30, CS only trials: p = 0.87). Extinction phase: Considering CR peak time, non-parametric ANOVA-type statistics revealed no significant main effect of Block (p = 0.43), Group (p = 0.74) or Group x Block interactions (p = 0.68). Download Figure 5-4, TIF file. [file enu-eN-NWR-0128-20-s06.tif]

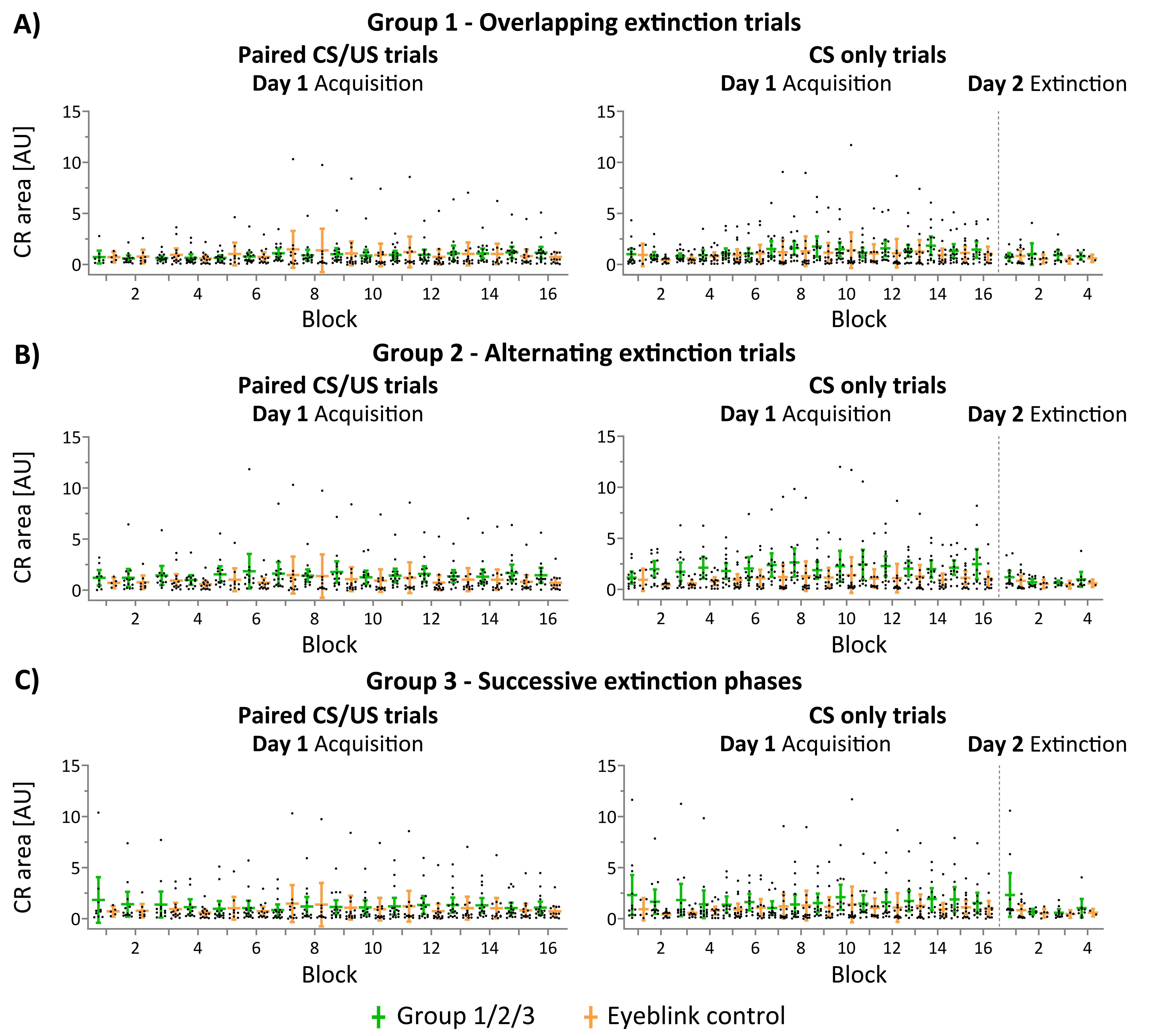

Supplement: Figure 5-5. — Eyeblink conditioning: CR area under the rectified EMG curve (Arbitrary Units – AUs). Baseline CR area was assessed in an interval of 100 ms prior US onset in each trial. Baseline area (corresponding to the CR duration) was subtracted from the CR area. In paired CS/US paired trials, CR area was assessed in the time window between CR onset and (up to) US onset because of frequent overlap with the UR. In CS only trials, CR area was assessed from CR onset to CR termination. Group mean CR areas and individual data are shown in the acquisition phase on day 1 (16 blocks à 8 trials corresponding to 5 CS/US trials and 3 CS only trials per block), and in the extinction phase on day 2 (4 blocks à 8 trials). (A) Group 1 (“overlapping extinction trials”, shown in green) vs. Group 5 (control, shown in yellow); (B) Group 2 (“alternating extinction trials”, shown in green) vs. Group 5 (control, shown in yellow); (C) Group 3 (“successive extinction phases”, shown in green) vs. Group 5 (control, shown in yellow). Horizontal lines represent mean values, vertical lines indicate 95% confidence intervals. Black dots show individual data points. Acquisition phase: In paired CS/US trials, non-parametric ANOVA-type statistics revealed no significant effects of Block (p = 0.42), Group (p = 0.0788) or Group x Block interactions (p = 0.91). In CS only trials, non-parametric ANOVA-type statistics revealed a significant effect of Group (F3 = 5.43; p = 0.0015), but no significant effect of Block (p = 0.23) or Group x Block (p = 0.36) interactions. Post-hoc pairwise comparisons revealed significantly increased CR area in CS only trials in Groups 1, 2 and 3 compared to the Group 5 (control) during the acquisition phase (least square means tests, all p values < 0.0231). Extinction phase: Non-parametric ANOVA-type statistics revealed no significant main effect of Block (p = 0.18), Group (p = 0.14) or Group x Block interactions (p = 0.73). Download Figure 5-5, TIF file. [file enu-eN-NWR-0128-20-s07.tif]

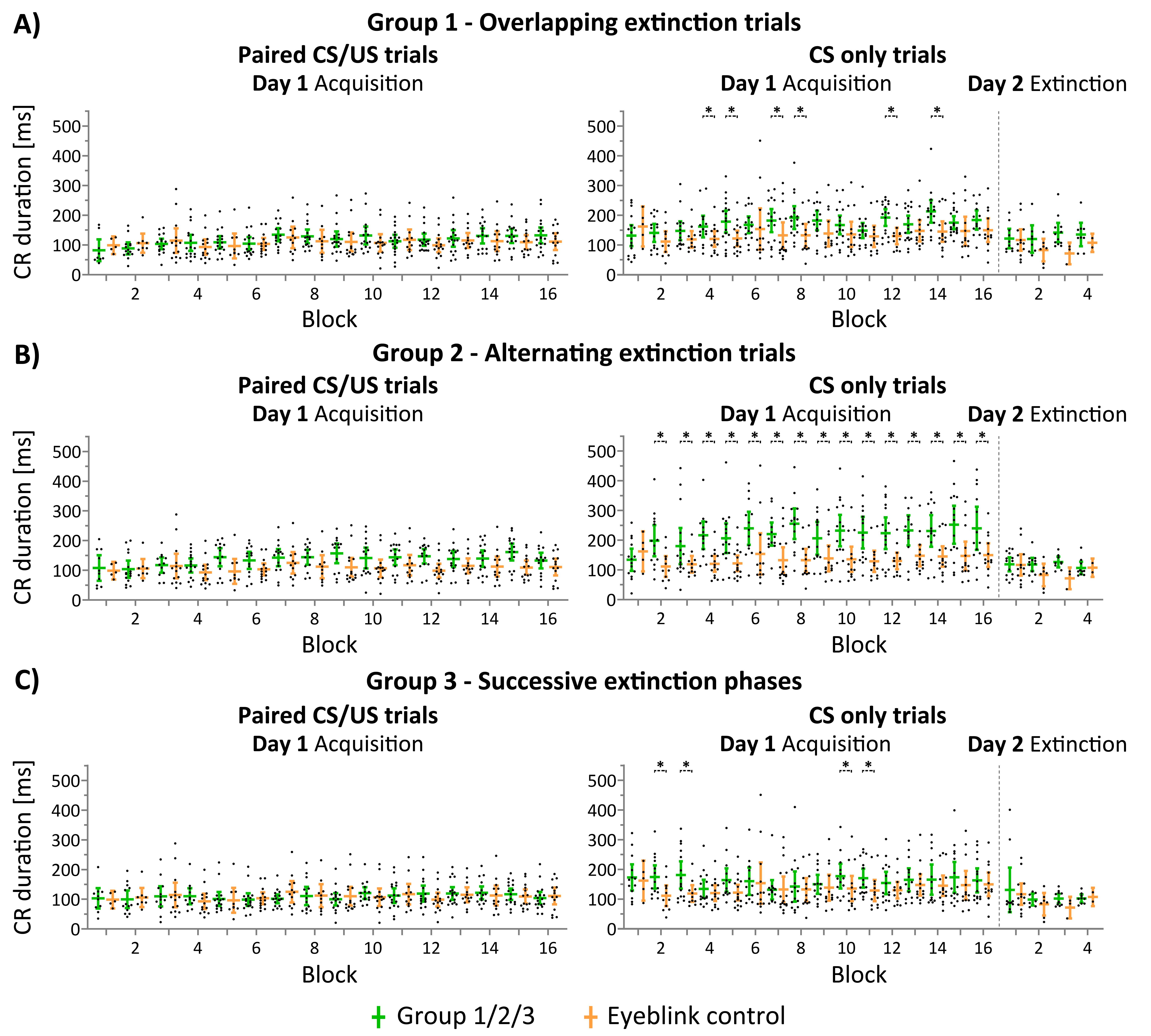

Supplement: Figure 5-6. — Eyeblink conditioning: CR duration. In paired CS/US paired trials, CR duration was assessed in the time window between CR onset and (up to) US onset because of overlap with the UR. In CS only trials, CR duration was assessed from CR onset to CR termination. Group mean CR durations and individual data are shown in the acquisition phase on day 1 (16 blocks à 8 trials corresponding to 5 CS/US trials and 3 CS only trials per block), and in the extinction phase on day 2 (4 blocks à 8 trials). (A) Group 1 (“overlapping extinction trials”, shown in green) vs. Group 5 (control, shown in yellow); (B) Group 2 (“alternating extinction trials”, shown in green) vs. Group 5 (control, shown in yellow); (C) Group 3 (“successive extinction phases”, shown in green) vs. Group 5 (control, shown in yellow). Horizontal lines represent mean values, vertical lines indicate 95% confidence intervals. Black dots show individual data points. * indicates significant differences between respective stimuli between Groups 1/2/3 and Group 5 (least square means tests, p values <0.05). Acquisition phase: In paired CS/US trials, non-parametric ANOVA-type statistics revealed no significant effects of Block (p = 0.28), Group (p = 0.44) or Group x Block interactions (p = 0.73). In CS only trials, non-parametric ANOVA-type statistics revealed a significant effect of Group (F3 = 8.09; p = 0.0002) and Group x Block interactions (F28.1 = 2; p = 0.0167), but no Block (p = 0.0708) effect. Post-hoc pairwise comparisons revealed significantly increased CR duration in CS only trials in Groups 1 and 2 compared to Group 5 (control) during the acquisition phase (least square means tests, all p values < 0.0055) but not Group 3 (p = 0.09). Extinction phase: Non-parametric ANOVA-type statistics revealed a significant main effect of Group (F2.79 = 3.89; p = 0.0157) but no Block (p = 0.49) or Group x Block interactions (p = 0.16). Post-hoc pairwise comparisons revealed significantly increased CR duration in Group 1 compa [file enu-eN-NWR-0128-20-s10.tif]
